# Facilitators and barriers to implementation of early intensive manual therapies for young children with cerebral palsy across Canada

**DOI:** 10.1186/s12913-025-12621-z

**Published:** 2025-04-04

**Authors:** Divya Vurrabindi, Alicia J. Hilderley, Adam Kirton, John Andersen, Christine Cassidy, Shauna Kingsnorth, Sarah Munce, Brenda Agnew, Liz Cambridge, Mia Herrero, Eleanor Leverington, Susan McCoy, Victoria Micek, Keith O. Connor, Kathleen O’ Grady, Sandra Reist-Asencio, Chelsea Tao, Stephen Tao, Darcy Fehlings

**Affiliations:** 1 Bloorview Research Institute, Rehabilitation Sciences Institute, Holland Bloorview Kids Rehabilitation Hospital, University of Toronto, Toronto, ON Canada; 2 University of Calgary, Alberta Children’s Hospital Research Institute, Calgary, AB Canada; 3 Department of Pediatrics, University of Calgary, Alberta Children’s Hospital Research Institute, Calgary, AB Canada; 4 Department of Pediatrics, University of Alberta, Glenrose Rehabilitation Hospital, Edmonton, AB Canada; 5 School of Nursing, Faculty of Health, Dalhousie University, Halifax, NS Canada; 6 Department of Occupational Science & Occupational Therapy, Rehabilitation Sciences Institute, Bloorview Research Institute, Teaching & Learning Institute, Holland Bloorview Kids Rehabilitation Hospital, University of Toronto, Toronto, ON Canada; 7 Institute of Health Policy, Management and Evaluation, Rehabilitation Sciences Institute, Bloorview Research Institute, Holland Bloorview Kids Rehabilitation Hospital, University of Toronto, Toronto, ON Canada; 8 CHILD-BRIGHT Network, Quebec, Canada; 9 Holland Bloorview Kids Rehabilitation Hospital, Toronto, ON Canada; 10 Alberta Children’s Hospital, Calgary, AB Canada; 11 Alberta Health Services, Calgary, AB Canada; 12 Glenrose Rehabilitation Hospital, Edmonton, AB Canada; 13 BC Children’s Hospital, Vancouver, BC Canada; 14 Department of Paediatrics, Bloorview Research Institute, Holland Bloorview Kids Rehabilitation Hospital, University of Toronto, 150 Kilgour Rd, Toronto, ON M4G 1R8 Canada

**Keywords:** Hemiplegic cerebral palsy, Consolidated framework for implementation research, Early intervention, Constraint induced movement therapy, Bimanual therapy, Implementation, Survey research

## Abstract

**Background:**

Cerebral Palsy (CP) is the most common childhood-onset motor disability. Play-based early intensive manual therapies (EIMT) is an evidence-based practice to improve long-term hand function particularly for children with asymmetric hand use due to CP. For children under two years old, this therapy is often delivered by caregivers who are coached by occupational therapists (OTs). However, why only a few Canadian sites implement this therapy is unclear. There is a need to identify strategies to support implementation of EIMT. The primary objective of this study was to identify the facilitators and barriers to EIMT implementation from the perspectives of (1) caregivers of children with CP (2), OTs and (3) healthcare administrators for paediatric therapy programs.

**Methods:**

The Consolidated Framework for Implementation Research (CFIR) was used to guide development of an online 5-point Likert scale survey to identify facilitators (scores of 4 and 5) and barriers (scores of 1 and 2) to implementation of EIMT. Three survey versions were co-designed with knowledge user partners for distribution to caregivers, OTs, and healthcare administrators across Canada. The five most frequently endorsed facilitators and barriers were identified for each respondent group.

**Results:**

Fifteen caregivers, 54 OTs, and 11 healthcare administrators from ten Canadian provinces and one territory participated in the survey. The majority of the identified facilitators and barriers were within the ‘Inner Setting’ CFIR domain, with ‘Structural Characteristics’ emerging as the most reported CFIR construct. Based on the categorization of the most frequently endorsed facilitators and barriers within the CFIR domains, the key facilitators to EIMT implementation included the characteristics of the intervention and establishing positive workplace relationships and culture. The key barriers included having workplace restrictions on EIMT delivery models and external influences (e.g., funding) on EIMT uptake.

**Conclusions:**

We identified key facilitators and barriers to implementing EIMT from a multi-level Canadian context. These findings will inform the next steps of designing evidence-informed and theory-driven implementation strategies to support increased delivery of EIMT for children under two years old with asymmetric hand use due to CP across Canada.

**Supplementary Information:**

The online version contains supplementary material available at 10.1186/s12913-025-12621-z.

## Background

Cerebral Palsy (CP) is the most prevalent childhood-onset physical disability affecting approximately 2 to 3 out of every 1,000 live births globally [1]. CP “describes a group of permanent disorders of the development of movement and posture, causing activity limitation, that are attributed to non-progressive disturbances that occurred in the developing fetal or infant brain” [2]. Depending on which part of the body is affected, CP can be further classified based on limb involvement to either quadriplegia (all four limbs), triplegia (one arm and two legs), diplegia (both legs), or hemiplegia (one arm and one leg). Hemiplegic cerebral palsy (HCP) is the most common subtype of CP at 38% [3] and is associated with asymmetric hand function. Other sub-types of CP, including triplegia and a subset of children with quadriplegia (approximately 40%) can also have asymmetric hand function [4].

International best clinical practice guidelines emphasize the need to provide early manual interventions for children between the ages of 0 to 2 years old with CP and asymmetric hand use to maximize function [5]. During the infant’s early developmental stages, there is a critical window for brain reorganization and neuroplasticity for upper extremity movements [6]. Executing early interventions to promote hand function during this window can positively alter developmental trajectories and augment long-term functional outcomes [5, 6].

The two most common intensive manual therapies for children with CP and asymmetric hand use are constraint induced movement therapy (CIMT) [7] and bimanual therapy [8]. CIMT involves constraining the preferred hand to increase use of the more affected hand, while bimanual therapy focuses on using both hands together to improve the ability to perform bimanual activities [9]. This study will use early intensive manual therapy or EIMT to encompass CIMT, bimanual therapy, or modified approaches. Early interventions for young children with CP under two years of age are typically play-based, with children engaged in enjoyable activities that elicit movement of the targeted limb. Administering high intensity therapy is related to improved manual outcomes [10, 11]. EIMT can be administered at a high intensity as caregivers are coached by therapists, with caregivers encouraged to do daily play sessions with their child [5].

A randomized controlled trial demonstrated that a modified home-based caregiver delivered CIMT, where occupational therapists (OTs) visited caregivers’ homes to coach them in administering the therapy, led to improved hand function among infants aged 3–8 months at high risk of hemiplegic CP [7]. Although there is limited research to date using bimanual therapy for children with CP under two years of age, one study, conducted by Chamudot et al. [12], compared a modified baby CIMT and bimanual therapy for children under two years and demonstrated equivalent positive outcomes. Furthermore, a small randomized controlled trial, comparing modified CIMT in home versus clinic settings for children under six years of age with hemiplegic CP, found significantly greater manual improvement in the home group, emphasizing the importance of a home-based therapy delivery environment [13].

There is currently limited access to EIMT programs for children with CP with asymmetric hand use under two years of age in Canada. This represents a science to practice gap, the reasons for which are poorly understood. Implementation science can play a crucial role in facilitating the effective integration of evidence-based knowledge into practical healthcare settings. Various implementation science frameworks can be used to assess factors that can either support or hinder implementation of an intervention or innovation of interest [14]. The Consolidated Framework for Implementation Research (CFIR), which has been recently updated, is a conceptual framework that provides a menu of constructs that can potentially facilitate or impede implementation [15, 16]. The CFIR is well suited to the aims of this research study, as it provides a practical approach to identifying potential barriers and facilitators in multiple contexts to implementing the intervention of interest.

Using the CFIR, we aimed to identify the barriers and facilitators to implementation of EIMT for children with CP under two years of age across Canada. The primary objective of the study was to determine the most common barriers and facilitators to implementation of EIMT for children under two years old with asymmetric hand use associated with CP by performing a quantitative online survey amongst three separate groups: caregivers of young children with CP, occupational therapists (OTs), and healthcare administrators. In addition, we postulated that there may be differences in perceived barriers and facilitators between caregivers and OTs with and without experience with EIMT that may inform future implementation strategies. Therefore, the secondary objective of the study was to identify any differences in facilitators and barriers between EIMT experienced caregivers and OTs and those who are non-experienced with EIMT.

## Methods

### Study design and conceptual framework

We used a cross-sectional quantitative five-point Likert scale survey design to analyze the barriers and facilitators to implementation of EIMT for children under two years of age with asymmetric hand use due to CP in Canada. The updated CFIR was used to guide survey development [16] to gain an extensive understanding of barriers and facilitators to implementation. The framework is composed of 48 constructs and 19 sub-constructs within five major domains: intervention characteristics, outer setting, inner setting, characteristics of the individuals involved, and the process of implementation [16]. Similar research has used the CFIR in cross-sectional survey designs to systematically capture potential facilitators and barriers to implementing evidence-based interventions [17, 18]. The Checklist for Reporting Results of Internet E-Surveys (CHERRIES) reporting guideline was used to guide survey development and reporting [19].

### Integrated knowledge translation

This study adopted an integrated knowledge translation approach [20]. Four caregivers with a child with CP, three OTs who work in pediatric settings, and two OT clinical practice or team leads were invited to join the research team and were engaged in protocol development, survey development, pilot testing, recruitment and survey distribution. A fifth caregiver joined the research team after pilot testing. For the knowledge user research partners, we administered the Public and Patient Engagement Evaluation Tool (PPEET) to obtain the knowledge users’ perceptions of their role in the study, and to self-assess their engagement [21]. The tool provides a set of criteria used to assess various aspects of public and patient engagement in healthcare research or service development. For our purposes, the survey was divided into four sections: Communication and Supports, Sharing Views, Impacts and Influence, and Final Thoughts. Participants answered 13 questions using a 5-point Likert scale, from “strongly disagree” to “strongly agree”. The survey was administered electronically via QUALTRICS (Qualtrics, Provo, UT) to all the knowledge user partners after survey data collection. Partners were asked to identify their group (caregiver, OT, lead), but no other identifying data were collected.

### Survey development

The online survey was developed using QUALTRICS software, Version [September, 2023]. The QUALTRICS XM platform collects survey responses once respondents complete the entire survey. The survey response data was safely stored in the software and only research team members (AJH or DV) had access to the data. Three survey versions were co-developed with knowledge user partners of the research team and tailored to each participant group: (1) parents/caregivers of children with CP; (2) OTs who treat children with CP; and (3) healthcare administrators who are responsible for managing OT programs that treat children with CP. Multiple virtual meetings facilitated by research team members AJH and DV were held for each knowledge user partner group to discuss and select constructs from the five CFIR domains (innovation characteristics, inner setting, outer setting, characteristics of individuals, and process). In each meeting, the name and definition of each CFIR construct was provided to knowledge users to determine which CFIR constructs were to be included in their respective survey versions. Knowledge users voted “yes” to include the construct in the survey or voted “no” if the construct was deemed irrelevant. If the majority of the group voted “yes” to a construct, it was included in the survey. Using the chosen CFIR constructs, research team members AJH and DV subsequently drafted five-point Likert type survey statements for each survey version. The full research team then reviewed, refined, and added any desired statements in meetings and asynchronously over email using shared cloud-based documents.

For the final survey design, respondents were instructed to rate the degree to which they agreed on the five-point Likert scale for each statement, with a sixth option of “N/A or Unsure” in the caregiver version and a statement of “I do not have the experience/knowledge to comment” in the OT and healthcare administrator versions. Options of “strongly agree” and “agree” indicated that the construct was a potential facilitator (factors that support or enable the implementation of EIMT therapies) and a response of “strongly disagree” or “disagree” indicated that the construct was a potential barrier (factors that hinder the ability to implement EIMT therapies) [17]. The option of “neutral” meant the respondent “neither agreed nor disagreed” with the statement, indicating that the construct was neither a barrier nor a facilitator. Most Likert survey statements were positively framed while twenty-four were negatively framed. Respondent demographics and therapy delivery history were also queried. Survey wording was evaluated using the Hemingway Editor and modified where necessary to meet a reading level of grade 8–10 [22].

Each survey version was piloted to contacts of knowledge user partners for further refinement. A total of 13 caregivers, 10 OTs and nine healthcare administrators or team leads completed the pilot surveys. Feedback from the pilot survey respondents was reviewed by knowledge user teams and suggestions were incorporated to finalize the survey versions. There were 41 questions for the caregiver survey version distributed across seven pages (screen) on the online survey (Appendix 1); 59 Likert survey questions for the OT survey version across ten pages (Appendix 2); and 63 questions for the healthcare administrator survey version across nine pages (Appendix 3^3^). The caregiver survey version included 23 constructs and 10 subconstructs from the CFIR, the OT survey included 34 CFIR constructs and 10 subconstructs, and the healthcare administrator survey included 41 CFIR constructs and 10 subconstructs. The implementation process domain was not included in the caregiver survey version, as our knowledge user partners did not deem this domain relevant. All five CFIR domains were included in the OT and healthcare administrator survey versions.

Respondent demographics and EIMT history-related questions included asking about their gender and race. In the caregiver survey, additional questions asked about the caregivers’ parental status (e.g., mother, father) and if they were co-caregivers or single caregivers in the home, financial supports, their child’s CP subtype, and their child’s history with EIMT. In the OT and healthcare administrator survey versions, additional questions asked about the years of experience in their roles, the availability of EIMT in their workplace, the types of EIMT practice models used, EIMT session frequency and their workplace. French and Arabic translations were made available for the caregiver survey version based on feedback from engaged knowledge user OTs. Respondents were able to review and change their answers (through a back button) and stop the survey at any point and return to where they left off at a later time/day. The survey questions were provided in the same order per version. “Forced response” features were added such that respondents were required to reply to all the Likert response statements. Moreover, adaptive question features were used to conditionally display certain questions based on the respondents’ answers to previous items. The online survey was open for an eight-month period (July 2023-January 2024).

Security features available in the QUALTRICS survey software were used to manage online bot attacks and fraudulent replies [23]. Security checklists were created by the research team members during the pre-data, data-collection, and post-data collection stages. Captcha, HTTP Cookies and software system IP address checks were added in the survey, to ensure that respondents were humans, and no bots or computer programs were written to spam the survey. Additionally, after completing the consent form, participants were presented with three open-text questions. They were prompted to enter (1) their email address (2), their Canadian province or territory of residence, and (3) how they heard about the study. A fourth question on a separate page asked respondents to re-enter their email address to ensure both matched [23, 24]. To identify potential fraudulent responses, researchers checked the timestamp (start and end time/date) of survey completion, validity of the respondents’ email address, and the location where the survey was completed based on postal code to ensure the survey was completed within Canada. Responses were excluded from the data set if the survey was completed in under two minutes [25], the respondents provided an invalid email address, or the survey system flagged duplicate entries from the same respondent. The exclusion criteria were used when identifying potential fraudulent responses, duplicate responses, as well as in instances of bot attacks (signaled by the sudden influx of 50 or more survey responses within a short period (i.e., 30 min) [23]. The survey software automatically emailed weekly reminders to non-responders for one month. The completion rate was then calculated based on the number of people who completed the entire survey divided by the number of people who agreed to complete the survey by completing the consent form (excluding bot responses).

### Survey participant characteristics and distribution

Across Canada, eligible participants were: (1) caregivers of a child six years old or younger with asymmetric hand use due to confirmed/suspected CP (2), OTs who treat children with CP and (3) healthcare administrators, team leads or people responsible for managing OT programs that provide rehabilitation services for children with CP. We aimed to recruit two caregivers from each Canadian province and territory, one from a rural location and one from an urban location. We aimed to recruit one OT and one healthcare administrator respondent from a tertiary workplace site (major centers providing comprehensive pediatric rehabilitation care) and one each from a community practice (providing services outside of a hospital setting including private practice or in schools) in each province and territory. Respondents received a $25 e-gift card after completing the survey. Knowledge user research partners shared the survey link via email to known potential respondents through snowball sampling (asking respondents to send the survey link to other potential respondents) and within known parent CP groups (closed parent Facebook groups). Snowball sampling was also used to share the survey link with OTs and managers working in different Canadian rehabilitation clinics. OT therapy organizations advertised the survey on their websites and service organizations (i.e. CP Canada and CP Alberta) advertised on their public social media platforms such as Facebook. We asked all organizations and clinics to circulate the survey internally among their members and family clients.

### Scoring and analysis

The facilitators and barriers survey responses were analyzed descriptively using the statistical software Jamovi (Version 2.5, Sept 2024). The survey response options were coded with numbers such that “strongly disagree” was coded as 1, “disagree” as 2, “neutral” 3, “agree” as 4, “strongly agree” as 5 and the sixth option as “6”. The negatively framed survey statements were reversed (i.e., scores of “4” and “5” were switched to “2” and “1”, respectively) on the Likert scale prior to tallying the frequencies. Aligned with the primary objective, the top five most frequently endorsed barriers and facilitators to implementation of intensive hand therapies were determined from each of the three participant survey versions based on the highest frequency responses (highest frequency of 1s and 2s or 4s and 5s for a statement respectively) [26, 27]. If more than five facilitator and barrier statements received the same frequency response rating, the statement that received a higher frequency response of a 5 versus a 4 and 1 versus a 2 was prioritized and reported respectively. The five most frequently endorsed facilitator and barrier survey statements were then organized into their respective CFIR domain, construct or sub-construct for reporting.

Similarities and differences in barriers and facilitators between the three participant groups were descriptively examined based on common (identified in each of the three survey participant groups) and participant-group specific CFIR constructs respectively. Given that a score of “6” (“I do not know”) may represent a barrier, an exploratory analysis was also done where the “6” scores were combined with scores of “1” and “2” to assess if this changed the top five barriers compared to frequency counts based solely on “1s” and “2s”. Following the survey analysis, the top five frequently endorsed facilitators and barriers across all three respondent groups were grouped into corresponding CFIR domains and respective constructs and/or sub-constructs within each domain.

To address the secondary objective, caregiver respondents were grouped into those who had received EIMT and those who had not, and OT respondents were categorized according to those who had experience delivering EIMT and those who did not. The differences in facilitators and barriers within the caregiver and OT subgroups were compared by calculating the mode score of all Likert survey responses and extracting statements with a ≥ 2 point mode score difference per subgroup. For the PPEET survey, the Likert scale response options were coded, and the median and interquartile ranges were calculated for each section of the survey.

## Results

### Demographics

**Fig. 1:**
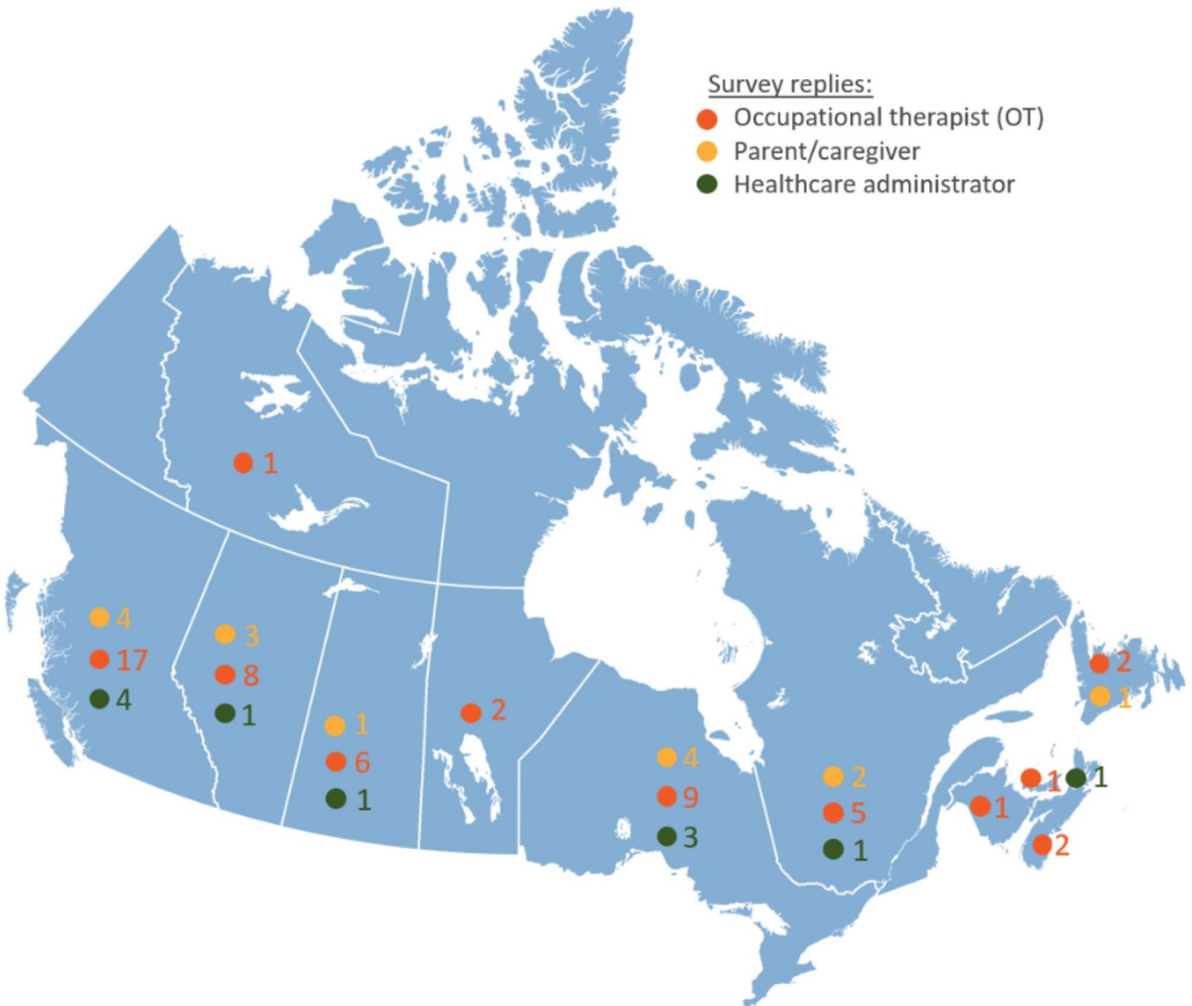
Distribution of survey replies from each participant group in Canada

In Qualtrics, 1004 potential participants consented to our study. Of those, 886 were identified as bot/blank responses, leaving 118 valid consent forms. Out of these 118, a total of 80 respondents (15 caregivers, 54 OTs, and 11 healthcare administrators) completed the survey across Canada resulting in a completion rate of 68% (80 out of 118). Respondent characteristics are summarized in Table 1. The survey responses were received from all 10 Canadian provinces and one territory as shown in Fig. 1.

**Table 1:** Characteristics of survey respondents

**Participant characteristics**	**Caregivers (*N*** **= 15)**	**OTs (*N*** **= 54)**	**Healthcare administrators (*N*** **= 11)**
	**N (%)**	**N (%)**	**N (%)**
**Sex**			
Female	13 (87)	53(98)	9 (82)
Prefer not to answer			1 (9)
**Race**			
Métis	1 (7)	0 (0)	0 (0)
White	12 (80)	41 (76)	9 (82)
Black	0 (0)	0 (0)	1 (9)
Southeast Asian	1 (7)	4 (7)	0 (0)
East Asian	0 (0)	7 (13)	0 (0)
Latin American	1 (7)	1 (2)	1 (9)
Prefer not to answer	0 (0)	1 (2)	0 (0)
**Clinic offering/caregivers receiving EIMT for children with CP < 2 years old**			
Yes	7 (47)	28 (52)	8 (73)
No	8 (53)	26 (48)	3 (27)
**Therapy assistants providing EIMT under the supervision of an OT**			
Yes		18 (33)	8 (73)
No		36 (67)	3 (27)
**Practice models used for EIMT of OT EIMT providers (*n*** **= 28) and healthcare administrators (*n*** **= 8)**			
OT coaches caregivers to delivery EIMT		23 (82)	6 (75)
OT hands-on delivers in-person therapy in clinic		14 (50)	5 (63)
OT hands-on delivers in-person therapy at clients’ home		7 (25)	2 (25)
OT delivers virtual sessions		8 (29)	3 (28)
More than one OT or assistant shares responsibility for therapy delivery for each child		6 (21)	2 (25)
**Frequency of sessions for subgroup of OT EIMT providers (*n*** **= 28) and healthcare administrators (*n*** **= 8) **			
Daily		14 (50)	2 (18)
1–2 times a week		9 (32)	
3–4 times a week		4(14)	2 (18)
5–6 times a week		4 (14)	
Unsure			4 (36)
**Place of work**			
Community Setting		33 (61)	7 (64)
Tertiary Center		17 (31)	3 (27)
Tertiary-Community Hybrid		4 (7)	1 (11)

The majority of the respondents were female and white 47% of caregivers, 52% of OTs (*n* = 28) and 73% of healthcare administrators had prior experience with EIMT. The majority of OTs that provided EIMT (*n* = 23, 82%) participated in a model where the OT coached caregivers to administer the therapy. There was also a mix of respondents with and without experience with EIMT who lived near tertiary centers or smaller communities demonstrating the ability to access EIMT across different locations/workplace settings.

Caregiver respondents were 12 mothers, two fathers, and one stepmother with the majority (*n* = 14, 93%) of the caregivers reporting they were a co-caregiver and 7% (*n* = 1) reporting they were a lone caregiver. The majority (*n* = 12, 80%) reported their child was diagnosed with CP by the age of one year. The rest of the caregivers (*n* = 3, 20%) reported their child was diagnosed by two years of age. Their child’s CP subtypes included hemiplegia (*n* = 8, 53%), triplegia (*n* = 1, 7%), quadriplegia (*n* = 2, 13%), 13% (*n* = 2) were unsure, and two respondents (13%) did not specify their child’s CP subtype. 87% (*n* = 13) of respondents reported they received OT services and most of those received OT before the age of one (*n* = 10, 67%). 47% of respondents (*n* = 7) reported their child had participated in EIMT before the age of two years. CIMT (*n* = 6, 40%) was reported as the intensive manual program provided, with one respondent (7%) reporting they were unsure which specific therapy was given. 60% (*n* = 9) of caregivers reported they received community support (e.g., funding for children with disabilities) for therapy services and 93% (*n* = 14) reported they had private health insurance.

Half of the OT respondents (*n* = 27, 50%) reported they had provided therapy for children with CP for at least 10 years and 74% (*n* = 40) had provided services for children with CP under the age of two years. Twenty-eight (52%) of the OT respondents had provided EIMT for children with CP under the age of two years. Out of the 28 OTs who provided EIMT, 15 OTs (54%) reported they used a model for EIMT where they coached caregivers and were also the primary therapy provider or conducted virtual therapy sessions, while eight OTs reported they strictly used a coaching model (29%). Of the 28 OTs that provide EIMT, nineteen (68%) reported using CIMT, four (14%) reported using both CIMT and bimanual therapy and five (18%) did not specify the specific protocol.

For the healthcare administrators, most had been in their current role for at least 10 years (*n* = 9, 82%). The majority (*n* = 9, 82%) reported the timeline between receiving a referral and a clients’ visit to the clinic was between one and 10 months. Eight out of the 11 healthcare administrators (73%) reported they supervise clinics that offer EIMT for children with CP under two years old. Six of these eight (75%) reported the most common practice model is the OT providing coaching sessions for caregivers to administer the therapy.

### Facilitators and barriers by respondent group

**Fig. 2:**
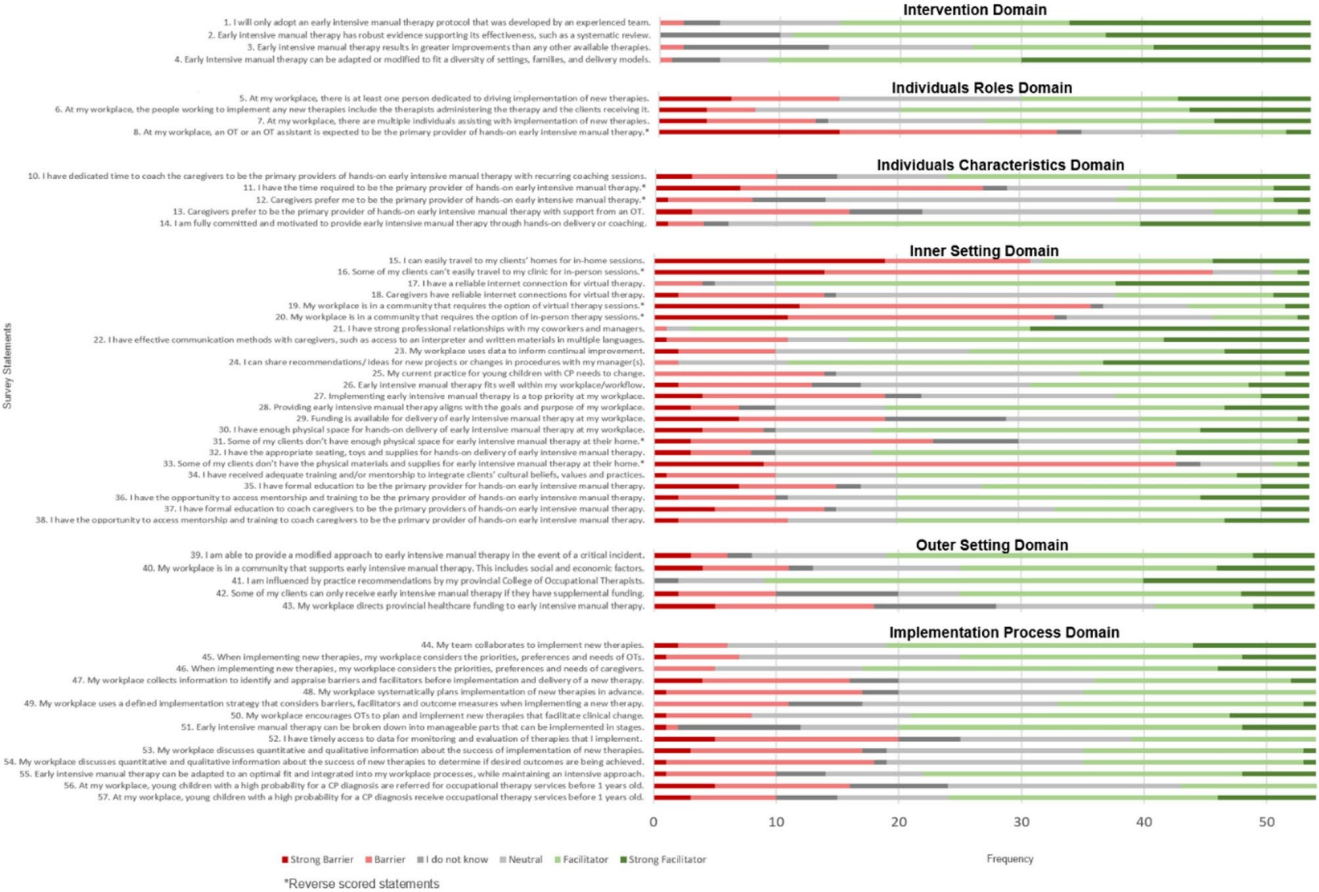
Frequency diagram of OT survey responses

The frequencies of each Likert statement survey response for the caregivers, OTs, and healthcare administrators survey versions can be found in Appendix 4, Fig. 2, and Appendix 5 respectively.

The five most frequently endorsed facilitators and barriers to implementation for caregivers, OTs and healthcare administrators are shown in Appendix 6, 7, and 8, respectively. Across all three respondent groups, 18 out of the total 30 top five frequently endorsed facilitators and barriers were from the ‘Inner Setting’ domain of the CFIR, four statements from the ‘Innovation’ domain, four from the ‘Outer Setting’ domain, three from the ‘Individuals’ domain and one from the ‘Implementation Process’ domain. The categorization of the most frequently endorsed facilitators and barriers into their respective CFIR domains are shown in Table 2. All three respondent groups shared a common facilitator (“I have reliable internet connect for virtual therapy”) and one barrier (“My workplace/circumstances require the option of in-person therapy sessions”) in the top five facilitator and barrier ratings. The OT and healthcare administrator groups shared a common facilitator (“I have strong professional relationships with my coworkers and managers”) and two barriers (“Some of my clients can’t easily travel to the clinic for in-person sessions” and “My workplace requires the option of virtual therapy sessions”). When the “6” scores were incorporated as potential barriers into the frequency assessment, only one of the caregiver barrier statements changed (“My decision to do a therapy is influenced by social media/advocacy groups” to “Intensive hand therapy is expensive”).

**Table 2:** Frequently endorsed facilitators and barriers identified by all three respondent groups categorized within their respective CFIR domains

CFIR Domain	Frequently Endorsed Facilitators and Barrier Likert Statements *Reverse scored statements
Intervention Domain (aspects of an intervention that may impact implementation success)	Caregiver Facilitator: I trust the people who recommended intensive hand therapy. OT Facilitator: Early Intensive manual therapy can be adapted or modified to fit a diversity of settings, families, and delivery models. Healthcare administrator Facilitator: Early intensive manual therapy has robust evidence supporting its effectiveness, such as a systematic review. Healthcare administrator Barrier: Early intensive manual therapy requires specialized training*.
Individuals Domain (individuals’ beliefs, knowledge, self-efficacy, and personal attributes that may affect implementation.)	Caregiver Facilitator: Recommendations from my physician, occupational therapist, or other clinician, influence whether my child participates in intensive hand therapy. OT Facilitator: At my workplace, an OT or therapist assistant is expected to coach caregivers to be the primary providers of hands-on early intensive manual therapy. OT Barrier: At my workplace, an OT or an OT assistant is expected to be the primary provider of hands-on early intensive manual therapy*.
Inner Setting Domain (characteristics of the implementing organization)	*Construct: Structural Characteristics* Caregiver/OT/Leads Facilitator: I have a reliable internet connection for virtual therapy. Caregiver Barrier: My therapy team includes more than one person who share hands-on delivery of intensive hand therapy for my child. Caregiver Barrier: More than one therapist teaches me to be the primary provider of hands-on intensive hand therapy. OT/ Healthcare administrator Barrier: Some of my clients can’t easily travel to my clinic for in-person sessions*. OT/ Healthcare administrator Barrier: My workplace is in a community that requires the option of virtual therapy sessions. Reasons could include travel time/cost or workplace policy*. OT/ Healthcare administrator barrier: My workplace is in a community that requires the option of in-person therapy sessions. Reasons could include limited bandwidth*. *Construct: Available Resources* OT Barrier: Some of my clients don’t have the physical materials and supplies for early intensive manual therapy at their home, such as appropriate seating or toys/objects*. Healthcare administrator Barrier: Some of my clients don’t have appropriate physical space to deliver early intensive manual therapy in their homes*. *Construct: Culture* Caregiver Facilitator: Occupational therapists and occupational therapist assistants are valued members of a child’s therapy team. Caregiver Facilitator: Caregivers of children with CP are valued members of a child’s therapy team. *Construct: Relational Connections* OT/Leads Facilitator: I have strong professional relationships with my coworkers and managers. Healthcare administrator Facilitator: New ideas for therapy are valued by my workplace team and leaders.
Outer Setting Domain (external influences on intervention implementation)	Caregiver Barrier: My decision to do a therapy is influenced by social media and/or advocacy groups. Caregiver Barrier: My child’s participation in intensive hand therapy depends on provincial health care funding*. Caregiver Barrier: My personal circumstances require in-person therapy*. OT Facilitator: I am influenced by practice recommendations by my provincial College of Occupational Therapists.
Implementation Process Domain (stages of implementation such as planning and evaluating, and the presence of key intervention stakeholders)	Healthcare administrator Facilitator: When implementing new therapies, my workplace considers the priorities, preferences and needs of caregivers.

### Differences in endorsed facilitators and barriers between EIMT experienced and non-experienced caregivers and OTs

Table 3 describes the difference in mode scores between EIMT experienced caregivers (*n* = 7) and non-experienced EIMT caregivers (*n* = 8) for their child and Table 4 describes the difference in mode scores between EIMT experienced OTs (*n* = 28) compared to non-experienced EIMTs OTs (*n* = 26).

**Table 3:** Survey statements with a ≥ 2 mode score difference between caregivers who received EIMT (*n* = 7) and caregivers who did not receive EIMT (*n* = 8) for children under two years of age

Survey Statement *Reverse scored statements	Experienced EIMT caregivers (*n* = 7)	Non-experienced EIMT caregivers (*n* = 8)
Intensive hand therapy is complicated to do* (Innovation Domain: Complexity).	4 (57%) (4 = disagree due to reverse scoring)	2 (50%) (2 = agree due to reverse scoring)
Intensive hand therapy is expensive* (Innovation Domain: Cost).	4 (29%) (4 = disagree due to reverse scoring)	2 (25%) (2 = agree due to reverse scoring)
My child’s participation in intensive hand therapy depends on a private health insurance plan* (Outer Setting Domain: Financing).	5 (43%) (5 = strongly disagree due to reverse scoring)	2 (25%) (2 = agree due to reverse scoring)
I have the necessary knowledge and skills to be the primary provider of hands-on intensive therapy to my child (Individuals Domain: Capability).	3 (29%)	1 (25%)
I have the necessary materials (toys, tables, chairs etc.) to provide intensive hand therapy (Inner Setting Domain: Physical Infrastructure).	5 (43%)	1 (25%)
I have enough physical space to provide intensive hand therapy at my home (Inner Setting Domain: Space).	5 (57%)	2 (25%)
I have the materials and supplies to deliver therapy at home, such as seating and toys (Inner Setting Domain: Materials & Equipment).	4 (43%)	2 (25%)
I have enough guidance and training to provide hands-on intensive hand therapy (Inner Setting: Access to Knowledge & Information).	4 (29%)	2 (38%)
My personal circumstances require the use of virtual therapy therapy* (Outer Setting Domain: Local Conditions).	4 (71%) (4 = disagree due to reverse scoring)	2 (75%) (2 = agree due to reverse scoring)
My decision to do a therapy is influenced by social media and/or advocacy groups (Outer Setting Domain: Societal Pressure).	1 (57%)	3

**Table 4:** OT survey statements with a ≥ 2 mode score difference between OT EIMT providers (*n* = 28) and OT EIMT non-providers (*n* = 26)

Survey Statements	OT EIMT providers (*n* = 28) (%)	OT EIMT non-providers (*n* = 26) (%)
Early intensive manual therapy fits well within my workplace/workflow (Inner Setting Domain: Adaptability).	4 (50%)	2 (33%)
Implementing early intensive manual therapy is a top priority at my workplace (Inner Setting Domain: Relative Priority).	4 (39%)	2 (42%)
My workplace is in a community that supports early intensive manual therapy. This includes social and economic factors, such as attitudes towards therapy and financial/time resources to attend therapy (Outer Setting Domain: Local Conditions).	4 (46%)	2 (25%)
I have timely access to data for monitoring and evaluation of therapies that I implement. This may include data from anecdotal feedback to validated outcome measures, on an individual or group level (Implementation Process Domain: Reflecting & Evaluating).	4 (36%)	2 (29%)
My workplace discusses quantitative and qualitative information about the success of new therapies to determine whether desired outcomes are being achieved (e.g., 2-point increase on COPM [Canadian Occupational Performance Measure]) (Implementation Process Domain: Innovation).	4 (43%)	2 (33%)

### PPEET survey

Eight out of the ten knowledge user partners from the research team (four caregivers, three OTs and one OT clinical practice lead) completed the PPEET survey. The results from the PPEET survey are included in Appendix 9. The survey results showed a median score of “strongly agree” (coded as “5”) across all sections, and interquartile range of zero for section two of the survey and an interquartile range of one for section one, three, and four of the survey.

## Discussion

**Fig. 3:**
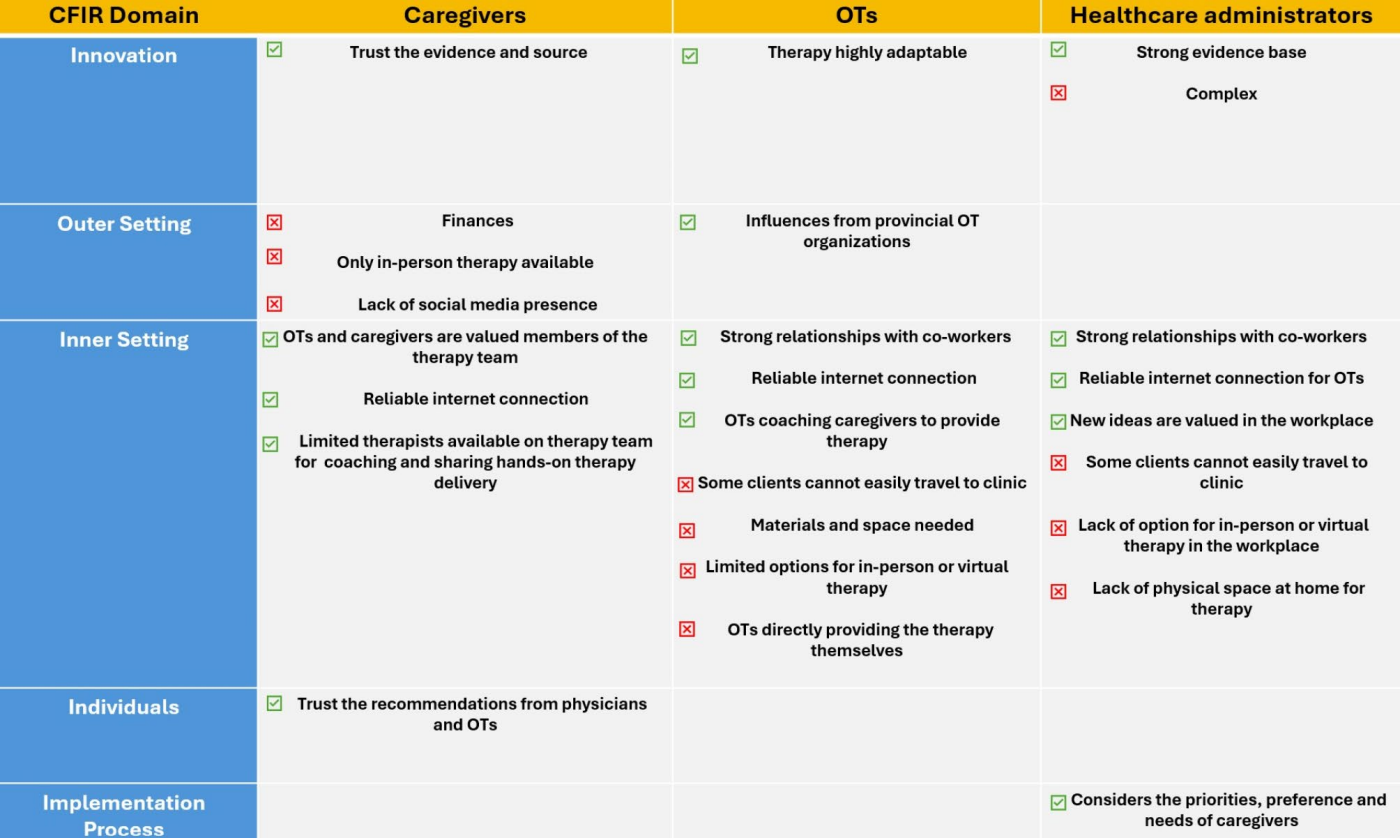
Key facilitators and barrier findings from caregiver, OT, and healthcare administrator respondent groups

In this study we identified the most frequently endorsed facilitators and barriers to implementation of EIMT for children with CP under two years of age from the perspectives of caregivers, OTs, and healthcare administrators. The most frequently endorsed facilitators and barriers spanned all five domains of the CFIR, with a majority of the statements identified from the ‘Inner Setting’ domain (Fig. 3). The rest of the endorsed constructs were identified within the ‘Innovation,’ ‘Individuals,’ ‘Outer Setting,’ and ‘Implementation Process’ domains, highlighting that the implementation of EIMT is impacted by individual as well as broader organizational/environmental contexts.

### Inner setting domain EIMT facilitators and barriers

The ‘Inner Setting’ domain captures the characteristics of the setting in which the innovation is implemented, either at an individual or organizational level [28]. Within this domain, the endorsed constructs included: ‘Structural Characteristics’, ‘Available Resources’, ‘Culture’, and ‘Relational Connections’. Most of the facilitators and barriers were within the ‘Structural Characteristics’ construct and were related to the delivery of EIMT. To begin, caregivers disagreed with the statement that their therapy team includes more than one person to share hands-on delivery of EIMT for their child (CFIR subconstruct: Work infrastructure) and that they have more than one therapist to teach them to be the primary provider of EIMT (CFIR subconstruct: Work infrastructure). These can be seen as barriers to implementation as caregivers may prefer the additional assistance of extra therapists or other caregivers in the family to mitigate the risk of provider burnout [29, 30]. Also, having additional coaches can alleviate time and scheduling constraints when one therapist becomes unavailable [31, 32]. Moreover, all three respondent groups endorsed that they have reliable internet connection for virtual therapy which is considered a facilitator to implementation of therapy interventions to minimize potential travelling costs for clients [33]. However, OTs and healthcare administrator respondents reported that their workplaces can mandate a specific model of EIMT delivery, either in-person or virtual (CFIR subconstruct: Information Technology Infrastructure). Employing fixed delivery models without flexibility can be a barrier to EIMT uptake as preferences may vary based on individual circumstances, such as families who cannot easily travel to a clinic for in-person sessions (which OTs and healthcare administrators endorsed) and those who may prefer in-person sessions.

The construct, ‘Available Resources’, which pertains to the extent to which implementation is influenced by resources, was also identified. Both OT and healthcare administrator respondents reported the barrier of families not having enough materials/equipment and physical space to carry out EIMT in the home environment (CFIR subconstruct: Materials & Equipment and Space). Reassuringly, in the context of EIMT, evidence from existing literature [7] and feedback from caregivers with firsthand experience of the therapy reports that it can be successfully administered with limited equipment and/or space in the home. The construct, ‘Culture’ (the shared values and beliefs in supporting the needs of deliverers) was identified. Caregivers agreed that OTs, therapist assistants as well as themselves are valued members of their child’s rehabilitation team (CFIR subconstruct: Deliverer Centeredness) which are considered facilitators to implementation [28, 34]. The construct ‘Relational Connections’ was also identified. Both healthcare administrators and OTs endorsed having strong professional relationships, along with healthcare administrators reporting that new ideas are valued by their workplace team and leaders. These findings highlight the importance of promoting a positive workplace culture encompassed by strong relationships where OTs, healthcare administrators, and caregivers are valued and work collaboratively to introduce and sustain new therapies such as EIMT. There is increasing recognition that the relational aspects of implementation science, including building trust and establishing rapport, are essential to facilitating implementation [35, 36]. Therefore, significant contextual factors within the inner setting including the characteristics and infrastructure of the workplace, the resources available in the home setting, the culture, and relationships within an organization, can all impact EIMT implementation efforts.

### Innovation domain EIMT facilitators and barriers

Four facilitators and barriers within the ‘Innovation’ domain were identified. This domain includes aspects of an intervention that may impact implementation success including evidence quality and strength, relative advantage, adaptability, complexity, and cost. First, caregivers reported trusting the people who recommend the therapy (CFIR construct: Early Intervention Source). Research highlights that the trustworthiness of the person recommending the intervention and their engagement with the individual are important for positive implementation outcomes [36]. Furthermore, the healthcare administrator respondents identified EIMT as having a strong evidence base (CFIR construct: Evidence Strength & Quality) such as support from systematic reviews [37] which is a facilitator for implementation [28]. OTs highlighted the adaptability of EIMT across various settings. The intervention’s adaptable nature can suit diverse settings which is also considered a strong facilitator to implementation (CFIR construct: Adaptability) [36, 38]. However, healthcare administrators reported that EIMT requires specialized training (CFIR construct: complexity). Interventions that are too complex can result in resistance to uptake within the intended clinical setting [39]. From these findings, we can take away that while the positive attributes of EIMT as an intervention can facilitate implementation, challenges such as the complexity of administering the therapy should be addressed when supporting these efforts.

### Individuals domain EIMT facilitators and barriers

The ‘Individuals’ domain captures characteristics of the individuals related to their professional roles and identities, skills and capabilities, which can impact implementation. In the context of EIMT, OT respondents reported that they are expected to coach caregivers to be the primary providers of hands-on EIMT (CFIR construct: Early Intervention Deliverers). However, OT respondents also endorsed that they are expected to be primary providers of hands-on EIMT (CFIR construct: Early Intervention Delivers). The practice model of coaching caregivers is a crucial component of caregiver-mediated interventions [40]. The practice of coaching also aligns with EIMT literature suggesting that optimal early intervention therapy outcomes are achieved through enhancing parent expertise and the generalization of rehabilitation activities into the child’s home environment [5, 13]. Although OTs can provide the therapy themselves, in the context of EIMT, the coaching model has been shown to enhance manual outcomes [5]. Therefore, to motivate and support caregivers to be the primary providers of EIMT, OTs can implement coaching strategies such as active engagement (i.e., opportunities for caregivers to practice skills), meaningful discussion, constructive feedback, and encouragement. These coaching strategies can help increase the caregiver’s motivation and self-efficacy thereby helping to sustain the intervention practice, as evidenced by the literature [40]. Caregivers reported being influenced by clinicians’ recommendations to do the therapy (CFIR construct: Mid-level leaders). Clinical leaders, serving as mid-level leaders in the CFIR framework, can help facilitate EIMT adoption. Therefore, the roles of both OTs and caregivers involved in delivering EIMT may influence the uptake of EIMT.

### Outer setting domain EIMT facilitators and barriers

Constructs were endorsed within the ‘Outer Setting Domain’ (the extent to which greater environmental contexts either support or hinder the ability to deliver the intervention). Caregivers reported dependence on provincial health care funding (CFIR construct: Financing). When “6” scores were included to assess barrier frequency, caregivers also reported that EIMT is expensive. The literature reports that insufficient personal finances can be a barrier to intervention uptake [32]. Also, it was found that caregivers’ personal circumstances can limit their access to EIMT (CFIR construct: Local Conditions). Previous research has highlighted that considering aspects of the caregivers’ circumstances including location, education, household income, and collecting sociodemographic data in therapy clinics needs to be prioritized when adapting caregiver interventions into practice [41, 42] and to enable equitable service provision. Interestingly, caregivers reported that the decision to do a therapy is not influenced by social media and/or advocacy groups (CFIR construct: Societal Pressure). It may be that social media is used as a source of health information [43], but not a factor in caregivers’ decision-making processes about their child’s therapy. Additionally, a research gap exists regarding effective strategies for leveraging social media in healthcare decision-making processes [44]. Further exploration is needed to understand the limited influence of social media as a facilitator or barrier. Of note, OTs reported being influenced by their provincial College of Occupational Therapists (CFIR construct: Policies & Law). Therefore, provincial organizations can play a role in supporting EIMT uptake. While modifying constructs in the ‘Outer Setting’ including provincial healthcare funding or exploring alternative equitable funding sources to support EIMT is challenging, promoting a model that is tailored to the caregivers’ circumstances may help increase uptake.

### Implementation process domain EIMT facilitators and barriers

The ‘Implementation Process’ domain, which highlights the stages of implementation such as planning, and evaluating, and the presence of key intervention stakeholders, had one endorsed facilitator. Healthcare administrators reported that they consider caregiver priorities, perspectives and needs when introducing new therapies such as EIMT (CFIR construct: Innovation Deliverers). Previous implementation science literature outlines that when staff members value and cater to the caregiver’s needs, implementation is supported [28, 32, 45]. Metz et al. [46] highlighted that effective and sustainable implementation of evidence-based practices is supported by bi-directional communication among implementation stakeholders including families and staff in a New York City’s public child welfare system study [46]. This underscores the significance of positive relational aspects serving as a strong facilitator to implementation and sustaining change [46].

### Differences in facilitators and barriers between EIMT experienced and non-experienced caregivers and OTs

Differences in facilitators and barriers within caregivers and OT subgroups who had experience with and without receiving or delivering EIMT were identified. In the subgroup of caregivers who received EIMT, they agreed EIMT was not complicated, was affordable, that they were knowledgeable/capable in EIMT, and had enough space and materials at home. The caregivers without EIMT experience disagreed with these same constructs. These findings highlight challenges unique to those who have not received EIMT. Therefore, learnings from these differences in the caregiver subgrouping suggest the possibility of partnering with EIMT experienced caregivers to support future implementation initiatives. Similarly, in the OT subgrouping, OTs offering EIMT affirmed its compatibility within their workplace schedules, their workplaces prioritizing EIMT implementation and providing opportunities to examine and evaluate therapy successes as facilitators. Conversely, OTs who did not provide EIMT disagreed with these same constructs. These findings highlight that OTs experienced with EIMT may not have encountered major challenges or overcame/solved issues, while those lacking prior experience are reporting significant barriers. A focused implementation strategy tailored to this OT subgroup (those without experience with EIMT) involving knowledge champions [47] (e.g., OTs experienced with EIMT) could be considered.

### Next steps

This study provides the necessary information to strategically identify, and design evidence informed, theory-driven implementation strategies to support increased delivery of EIMT. Next steps of this research will build from survey results using a mixed-methods sequential explanatory design. The multidisciplinary research team will first select modifiable barriers and facilitators identified from the survey results. The Expert Recommendations for Implementation Change taxonomy will be used in mapping the identified modifiable facilitators and barriers [48]. Semi-structured interviews with caregivers, occupational therapists, and healthcare administrators will be conducted to further explore barriers, facilitators, and potential implementation strategies. Finally, a co-design session with the three participant groups will be conducted to prioritize and refine a final list of implementation strategies. These strategies will be designed in partnership with our knowledge user research partners to ensure their needs and goals are met and strategies are tailored to each group. The findings from this study may be valuable to Canadian health system leaders as well as implementation practitioners to inform their implementation efforts, particularly those interested in implementing EIMT or similar caregiver-mediated interventions. The success of implementation strategies will need to be studied in future implementation practice research.

### Strengths and limitations

Our study has several strengths. First, our study used the CFIR which provides a framework to ensure a multi-system perspective to assessing the facilitators and barriers to implementing EIMT across Canada. Moreover, this study invited knowledge users to be an integral part of the research team and were actively involved in key components of the research study including survey development and recruitment. The knowledge user partners reported they were highly engaged in the project on the PPEET. The integrated knowledge translation approach has demonstrated improved outcomes in research related to improving the health of Canadians and the Canadian healthcare system [20]. Our study also has limitations. While we achieved broad representation across Canada and a mix of OT and health care administrators from community and tertiary settings, some provinces and the northern territories were under-represented. As a result, our sample is not evenly distributed, making it challenging to capture provincial differences in barriers and facilitators. Moreover, the representation from the northern regions was limited to just one respondent and the majority of our survey respondents were White. Consequently, we were unable to capture diverse cultural perspectives on the facilitators and barriers to EIMT. Future research is required to better understand experiences of historically disadvantaged groups, including Indigenous peoples. Implementation context is very important to understanding barriers and facilitators, therefore our findings may not be applicable in other contexts such as low- and middle-income countries. Our study provides a strong example of how future needs assessments can be conducted in different contexts.

## Conclusion

This study used implementation science to understand the perceived facilitators and barriers to implementation of EIMT for children under two with CP from the perspectives of caregivers, OTs and healthcare administrators. The majority of the facilitators and barriers were identified within the ‘Inner Setting’ domain of the CFIR, highlighting the need to prioritize developing and tailoring strategies to this domain to increase EIMT adoption. Offering various EIMT delivery models that consider differing caregiver circumstances, fostering positive workplace values, and supporting relationships between workplace staff as well as with caregivers is important. Additionally, taking advantage of the strengths of EIMT, including its strong evidence base and adaptability as an intervention, could encourage greater adoption and utilization. Strategies that utilize caregivers and OTs with firsthand experience in EIMT as knowledge champions may also help to address barriers in caregivers and OTs without experience in EIMT. Informed by our findings, the next steps of implementation strategy design hold promise in increasing the delivery and uptake of EIMT for young children with CP across Canada.

## Supplementary Information

Supplementary Material 1: Appendix 1. Caregiver Survey Version.

Supplementary Material 2: Appendix 2. Occupational Therapist Survey Version.

Supplementary Material 3: Appendix 3. Healthcare administrator Survey Version.

Supplementary Material 4: Appendix 4. Frequency Diagram of Caregiver Survey Responses.

Supplementary Material 5: Appendix 5. Frequency Diagram of Healthcare Administrators Survey Responses.

Supplementary Material 6: Appendix 6. Frequency diagram of Caregiver respondents (*n* = 15) of top five most frequently endorsed facilitators (top) and barriers (bottom) to implementation reported.

Supplementary Material 7: Appendix 7. Frequency diagram of OT respondents (*n* = 54) of top five most frequently endorsed facilitators (top) and barriers (bottom) to implementation reported.

Supplementary Material 8: Appendix 8. Frequency diagram of healthcare administrator respondents (*n* = 11) of top five most frequently endorsed facilitators (top) and barriers (bottom) to implementation reported.

Supplementary Material 9: Appendix 9. Results from the PPEET Survey (*n* = 4 Caregivers, *n* = 3 OTs, *n* = 1 OT clinical practice lead).

## Data Availability

The datasets used and/or analysed during the current study are available from the corresponding author on reasonable request.

## Abbreviations

CP: Cerebral palsy
HCP: Hemiplegic cerebral palsy
CIMT: Constraint induced movement therapy
CHERRIES: Checklist for Reporting Results of Internet E-Surveys
OT: Occupational therapist
EIMT: Early intensive manual therapy
CFIR: Consolidated Framework for Implementation Research
PPEET: Patient Partner Engagement Evaluation Tool
