# Supplementary material for: Facilitators and barriers to implementation of early intensive manual therapies for young children with cerebral palsy across Canada
Source: BMC Health Serv Res. 2025 Apr 4;25:503. doi: 10.1186/s12913-025-12621-z (PMC11971912; doi:10.1186/s12913-025-12621-z)
Supplement: Supplementary file 1 — Supplementary Material 1: Appendix 1. Caregiver Survey Version. [file 12913_2025_12621_MOESM1_ESM.docx]

**Caregiver Survey Version**

Welcome to Part One.

In Part One of the survey, we ask about things that influence participation in an intensive therapy program for children with cerebral palsy (CP) under 2 years old. We are focusing on hand therapy. This may be constraint induced movement therapy (CIMT), bimanual therapy, or another type of therapy that focuses on your child’s weaker/less preferred hand. An intensive therapy program involves practice daily or many times per week. Caregivers may deliver therapy, caregivers may be supported by a therapist, and/or a therapist may deliver therapy. Appointments with therapists may be in-person, at a clinic or the child’s home, or virtual over video call.

Part One survey questions that have six response options. If the statement doesn’t apply to your situation (not applicable) or you’re unsure, you can select “N/A or Unsure”.

| **PART ONE: Statements** | Response | | | | | |
| --- | --- | --- | --- | --- | --- | --- |
|  | Strongly Disagree | Disagree | Neutral | Agree | Strongly Agree | N/A or Unsure |
| I trust the people who recommended intensive hand therapy. |  |  |  |  |  |  |
| Intensive hand therapy is better than any other available therapies. |  |  |  |  |  |  |
| Intensive hand therapy can be modified for where the therapy happens, like my home or the occupational therapist’s clinic. |  |  |  |  |  |  |
| Intensive hand therapy is complicated to do. |  |  |  |  |  |  |
| Intensive hand therapy has clear steps and helpful documents. |  |  |  |  |  |  |
| Intensive hand therapy is expensive. |  |  |  |  |  |  |
| Recommendations from my physician, occupational therapist, or other clinician, influence whether my child participates in intensive hand therapy |  |  |  |  |  |  |
| An Occupational therapist should be the primary provider of hands-on intensive therapy to my child |  |  |  |  |  |  |
| I should be the primary provider of intensive hand therapy. |  |  |  |  |  |  |
| I have the necessary knowledge and skills to be the primary provider of hands-on intensive therapy to my child. |  |  |  |  |  |  |
| I have the time to learn how to provide hands-on intensive therapy to my child. |  |  |  |  |  |  |
| My child’s occupational therapist has time to teach me to be the primary provider of hands-on intensive therapy. |  |  |  |  |  |  |
| I am committed and motivated to be the primary provider of hands-on intensive therapy to my child. |  |  |  |  |  |  |
| Occupational therapists are committed and motivated to teach me to provide intensive hand therapy to my child. |  |  |  |  |  |  |
| I have the necessary materials (toys, tables, chairs etc.) to provide intensive hand therapy. |  |  |  |  |  |  |
| I have reliable internet connection for virtual therapy. |  |  |  |  |  |  |
| My therapist(s) have reliable internet connections for virtual therapy. |  |  |  |  |  |  |
| My therapy team includes more than one person who share hands-on delivery of intensive hand therapy for my child. |  |  |  |  |  |  |
| More than one therapist teaches me to be the primary provider of hands-on intensive hand therapy. |  |  |  |  |  |  |
| I have positive relationships with my therapist(s). |  |  |  |  |  |  |
| Caregivers of children with CP are valued members of a child's therapy team. |  |  |  |  |  |  |
| Occupational therapists and therapy assistants are valued members of a child's therapy team. |  |  |  |  |  |  |
| My child’s therapist(s) believe in continuously improving the therapy they provide. |  |  |  |  |  |  |
| Immediate resources and therapies are available for young children with CP. |  |  |  |  |  |  |
| Intensive hand therapy fits well into my family's schedule. |  |  |  |  |  |  |
| Intensive hand therapy is a top priority for my child, more than other therapies. |  |  |  |  |  |  |
| My clinic has the necessary funding to offer intensive hand therapy. |  |  |  |  |  |  |
| I have enough physical space to provide intensive hand therapy at my home. |  |  |  |  |  |  |
| My therapists have enough physical space for hands-on delivery of intensive hand therapy at their clinic. |  |  |  |  |  |  |
| I have the materials and supplies to deliver therapy at home, such as seating and toys. |  |  |  |  |  |  |
| I have enough guidance and training to provide intensive hand therapy. |  |  |  |  |  |  |
| The guidance and training to deliver intensive hand therapy can be provided in my preferred language. |  |  |  |  |  |  |
| The circumstances of my community support intensive therapy. Circumstances include social and economic factors, such as attitudes towards therapy and financial/time resources to attend therapy. |  |  |  |  |  |  |
| My personal circumstances require the use of virtual therapy. |  |  |  |  |  |  |
| My personal circumstances require in-person therapy. |  |  |  |  |  |  |
| Policies and laws such as the Child Youth and Family Services Act, influence my child’s participation in intensive hand therapy. |  |  |  |  |  |  |
| My child’s participation in intensive hand therapy depends on a private health insurance plan. |  |  |  |  |  |  |
| My child’s participation in intensive hand therapy depends on Jordan’s Principle (government funding for First Nations children). |  |  |  |  |  |  |
| My child’s participation in intensive hand therapy depends on provincial health care funding. |  |  |  |  |  |  |
| My decision to do a therapy is influenced by social media and/or advocacy groups. |  |  |  |  |  |  |
| I am comfortable with my clinic being compensated for providing therapy services that they have recommended. |  |  |  |  |  |  |

Thank you for completing Part One. Do you have any feedback to share?

________________________________________________________

**PART TWO: Demographics**

Q1. What are the first three digits of your postal code?
______________________________________________

Q2. How old is your child who has a less-preferred hand?

- Years__________________________________________
- Months __________________________________________________

Q3. Has your child been diagnosed with cerebral palsy?

- Yes
- No

Display This Question:

If Has your child been diagnosed with cerebral palsy? = Yes

Q4. At what age was your child diagnosed with cerebral palsy?

- Years __________________________________________________
- Months __________________________________________________

Display This Question:

If Has your child been diagnosed with cerebral palsy? = Yes

Q5. What type of cerebral palsy does your child have?

- Hemiplegia (one body side affected)
- Triplegia (both legs and one hand/arm affected)
- Quadriplegia (both legs and both hand/arms affected)
- Not sure

Q6. Has your child received any other diagnoses?

- Yes
- No

Display This Question:

If Has your child received any other diagnoses? = Yes

Q7. Please list other diagnoses.
_____________________________________________________

Q8. Has your child received occupational therapy (hand therapy)?

- Yes
- No
- My child does not need hand therapy

Display This Question:

If Has your child received occupational therapy (hand therapy)? = Yes

Q9. How old was your child when they first received occupational therapy?

- Years __________________________________________________
- Months __________________________________________________

Display This Question:

If Has your child received occupational therapy (hand therapy)? = Yes

Q10. Did your child participate in an intensive program with multiple therapy times per week before the age of two years?

- Yes
- No

Display This Question:

If Did your child participate in an intensive program with multiple therapy times per week before th... = Yes

Q11. What was the program? Select all that apply.

- Constraint Induced Movement Therapy (CIMT), with one-handed activities for your child's less preferred hand. Their preferred hand is "hidden" using a mitt or something similar.
- Bimanual Therapy, with two-handed activities where both hands work together.
- Not sure

Display This Question:

If Has your child received occupational therapy (hand therapy)? = No

Or Has your child received occupational therapy (hand therapy)? = My child does not need hand therapy

Q12. Was your child offered hand therapy before two years old?

- Yes
- No

Display This Question:

If Was your child offered hand therapy before two years old? = Yes

Q13. Why did your child not participate?

Q14. Have you been a caregiver for your child since his or her birth?

- Yes
- No

Display This Question:

If Have you been a caregiver for your child since his or her birth? = No

Q15. How long have you been a caregiver to this child?

- Years __________________________________________________
- Months __________________________________________________

Q16. What is your current caregiver status?

- Mother
- Father
- Grandparent
- Foster Parent
- Other (please specify): __________________________________________________

Q17. What best describes your caregiver status throughout your child’s life up to now?

- Lone caregiver in the home
- Co-caregiver in the home (e.g., partner or adult family member also cares for the child)
- Other (please specify): __________________________________________________

Q18. How many children do you have?

- 1
- 2
- 3
- 4+

Q19. What is your marital status?

- Married/common-law
- Divorced/separated – currently single
- Divorced/separated – currently remarried/common-law
- Single

Q20. Do you receive community support for services (e.g., funding for children with disabilities)?

- Yes
- No

Display This Question:

If Do you receive community support for services (e.g., funding for children with disabilities)? = Yes

Q21. Please specify the community support you receive:
_______________________________________________________________

Q22. Do you have private health insurance?

- Yes
- No

Q23. Do you receive support from Jordan’s Principle for First Nations children?

- Yes
- No

*The remaining questions in Part Two are included to ensure that we capture diverse voices in this research. You have the option to select "Prefer not to answer" for these questions.*

Q24. What is the highest level of schooling you have obtained?

- Indigenous knowledge keeper
- Grade school certificate
- High school certificate
- College certificate or diploma
- Bachelor’s degree
- Master’s, doctorate, or professional (e.g., law, dentistry, pharmacy) degree
- Prefer not to answer

Q25. What was your household's total gross income (before taxes end deductions) last year (2022)?

- < $30,000
- $30,000 to $70,000
- $71,000 to $110,000
- $111,000 to $150,000
- >$151,000
- Prefer not to answer

Q26. Do you identify as a Racialized person?

- Yes
- No
- Prefer not to answer

Q27. Which of the following best describes you? 
Select all that apply.

- First Nations
- Métis
- Inuit
- Black
- East Asian (Chinese, Korean, Japanese)
- Latin American
- Middle Eastern (Arab, West Asian (e.g., Iranian, Afghan))
- South Asian (e.g., East Indian, Pakistani, Sri Lankan)
- Southeast Asian (Filipino, Vietnamese, Cambodian, Laotian, Thai)
- White
- Do not know
- Other please specify: __________________________________________________
- Prefer not to answer

Q28. What language(s) do you speak on a regular basis at home?
Select all that apply.

- English
- French
- Other (please specify): __________________________________________________
- Prefer not to answer

Q29. Do you self-identify as a person with a disability?

- Yes
- No
- Prefer not to answer

Display This Question:

If Do you self-identify as a person with a disability? = Yes

Q30. Please indicate the types(s) of disability you have:

- Visible
- Non-visible
- Both
- Prefer not to answer

Q31. How do you identify with regards to your gender?
Select all that apply.

- Woman
- Man
- Non-Binary, Gender Queer, or a similar term
- Two-spirit
- Transwoman
- Transman
- Other (please specify): __________________________________________________
- I do not identify with a gender
- Prefer not to answer

Q32. How do you identify with regards to your sexual orientation?
Select all that apply.

- Heterosexual/Straight
- Asexual
- Bisexual
- Two-spirit
- Gay
- Lesbian
- Queer
- Other (please specify): __________________________________________________
- Prefer not to answer

Q33. Please indicate which applies to you:

- I was born in Canada
- I was born in another country and then immigrated to Canada
- Prefer not to answer

Q34. Please indicate which applies to your child:

- My child was born in Canada
- My child was born in another country and then immigrated to Canada
- Prefer not to answer

Q35. Thank you for completing Part Two, which included questions about you.

Do you have any feedback about the questions in Part Two?
_____________________________________________________________

Q36. Thank you for participating in the survey. Please share any feedback here.

_____________________________________________________________
