# Supplementary material for: Facilitators and barriers to implementation of early intensive manual therapies for young children with cerebral palsy across Canada
Source: BMC Health Serv Res. 2025 Apr 4;25:503. doi: 10.1186/s12913-025-12621-z (PMC11971912; doi:10.1186/s12913-025-12621-z)
Supplement: Supplementary file 2 — Supplementary Material 2: Appendix 2. Occupational Therapist Survey Version. [file 12913_2025_12621_MOESM2_ESM.docx]

**Occupational Therapist Survey Version**

Welcome to Part One.

In Part One of the survey, we ask about things that influence delivery of an intensive hand therapy program for children under 2 years old. We are focusing on hand therapy. This may be constraint induced movement therapy (CIMT), bimanual therapy, or another type of therapy thar focuses on the child’s weaker/less-preferred hand. An intensive therapy program involves practice daily or many times per week. Caregivers may deliver therapy, therapists may support caregivers, or a therapist may deliver therapy. Visits with OTs may be in-person, at a clinic or the child’s home, or virtual over video call. We are asking about your current practice.

Part One survey questions are statements that have six response options.

| **PART ONE: Statements** | Response | | | | | |
| --- | --- | --- | --- | --- | --- | --- |
|  | Strongly Disagree | Disagree | Neutral | Agree | Strongly Agree | I do not have the experience/ knowledge to comment |
| I will only adopt an early intensive manual therapy protocol that was developed by a reputable team. |  |  |  |  |  |  |
| Early intensive manual therapy has robust evidence supporting its effectiveness, such as a systematic review. |  |  |  |  |  |  |
| Early intensive manual therapy results in greater improvements than any other available therapies. |  |  |  |  |  |  |
| Early intensive manual therapy can be adapted or modified to fit a diversity of settings, families, and delivery models. |  |  |  |  |  |  |
| At my workplace, there is at least one person dedicated to driving implementation of new therapies, and/or facilitating implementation decisions of any new therapies. |  |  |  |  |  |  |
| At my workplace, the people working to implement any new therapies include the therapists administering the therapy and the clients receiving it. |  |  |  |  |  |  |
| At my workplace, there are multiple individuals assisting with implementation of new therapies. |  |  |  |  |  |  |
| At my workplace, an OT or a therapy assistant is expected to be the primary provider of hands-on early intensive manual therapy. |  |  |  |  |  |  |
| At my workplace, an OT or therapy assistant is expected to coach caregivers to be the primary providers of hands-on early intensive manual therapy. |  |  |  |  |  |  |
| I have dedicated time to coach the caregivers to be the primary providers of hands-on early intensive manual therapy with recurring coaching sessions. (Note: This can include support from your team, e.g., another OT or therapist assistant completing some visits.) |  |  |  |  |  |  |
| I have the time required to be the primary provider of hands-on early intensive manual therapy. (Note: This can include support from your team, e.g., another OT or therapist assistant completing some visits.) |  |  |  |  |  |  |
| Caregivers prefer me to be the primary provider of hands-on early intensive manual therapy. |  |  |  |  |  |  |
| Caregivers prefer to be the primary provider of hands-on early intensive manual therapy with support from an OT. |  |  |  |  |  |  |
| I am fully committed and motivated to provide early intensive manual therapy through hands-on delivery or coaching. |  |  |  |  |  |  |
| I can easily travel to my clients’ homes for in-home sessions. |  |  |  |  |  |  |
| Some of my clients can’t easily travel to my clinic for in-person sessions. |  |  |  |  |  |  |
| I have a reliable internet connection for virtual therapy. |  |  |  |  |  |  |
| Caregivers have reliable internet connections for virtual therapy. |  |  |  |  |  |  |
| My workplace is in a community that requires the option of virtual therapy sessions. Reasons could include travel time/cost or workplace policy. |  |  |  |  |  |  |
| My workplace is in a community that requires the option of in-person therapy sessions. Reasons could include limited bandwidth. |  |  |  |  |  |  |
| I have strong professional relationships with my coworkers and managers. |  |  |  |  |  |  |
| I have effective communication methods with caregivers, such as access to an interpreter and written materials in multiple languages. |  |  |  |  |  |  |
| My workplace uses data to inform continual improvement. |  |  |  |  |  |  |
| I can share recommendations/ ideas for new projects or changes in procedures with my manager(s). |  |  |  |  |  |  |
| My current practice for young children with CP needs to change. |  |  |  |  |  |  |
| Early intensive manual therapy fits well within my workplace/workflow. |  |  |  |  |  |  |
| Implementing early intensive manual therapy is a top priority at my workplace. |  |  |  |  |  |  |
| Providing early intensive manual therapy aligns with the goals and purpose of my workplace. This refers to hands-on delivery and/or coaching caregivers. |  |  |  |  |  |  |
| Funding is available for delivery of early intensive manual therapy at my workplace. |  |  |  |  |  |  |
| I have enough physical space for hands-on delivery of early intensive manual therapy at my workplace. |  |  |  |  |  |  |
| Some of my clients don’t have enough physical space for early intensive manual therapy at their home. |  |  |  |  |  |  |
| I have the appropriate seating, toys and supplies for hands-on delivery of early intensive manual therapy, such as appropriate seating or toys/objects. |  |  |  |  |  |  |
| Some of my clients don’t have the physical materials and supplies for early intensive manual therapy at their home, such as appropriate seating or toys/objects. |  |  |  |  |  |  |
| I have received adequate training and/or mentorship to integrate clients' cultural beliefs, values and practices. |  |  |  |  |  |  |
| I have formal education to be the primary provider for hands-on early intensive manual therapy. |  |  |  |  |  |  |
| I have the opportunity to access mentorship and training to be the primary provider of hands-on early intensive manual therapy. |  |  |  |  |  |  |
| I have formal education to coach caregivers to be the primary providers of hands-on early intensive manual therapy. |  |  |  |  |  |  |
| I have the opportunity to access mentorship and training to coach caregivers to be the primary provider of hands-on early intensive manual therapy. |  |  |  |  |  |  |
| I am able to provide a modified approach to early intensive manual therapy in the event of a critical incident, like a global pandemic or change in political leadership. |  |  |  |  |  |  |
| My workplace is in a community that supports early intensive manual therapy. This includes social and economic factors, such as attitudes towards therapy and financial/time resources to attend therapy. |  |  |  |  |  |  |
| I am influenced by practice recommendations by my provincial College of Occupational Therapists. |  |  |  |  |  |  |
| Some of my clients can only receive early intensive manual therapy if they have supplemental funding such as private health insurance plans, community funding, or support from Jordan’s Principle. This refers to hands-on delivery and/or coaching caregivers. |  |  |  |  |  |  |
| My workplace directs provincial healthcare funding to early intensive manual therapy. |  |  |  |  |  |  |
| My team collaborates to implement new therapies. |  |  |  |  |  |  |
| When implementing new therapies, my workplace considers the priorities, preferences and needs of OTs. |  |  |  |  |  |  |
| When implementing new therapies, my workplace considers the priorities, preferences and needs of caregivers. |  |  |  |  |  |  |
| My workplace collects information to identify and appraise barriers and facilitators before implementation and delivery of a new therapy. |  |  |  |  |  |  |
| My workplace systematically plans implementation of new therapies in advance. |  |  |  |  |  |  |
| My workplace uses a defined implementation strategy that considers barriers, facilitators and outcome measures when implementing a new therapy. |  |  |  |  |  |  |
| My workplace encourages OTs to plan and implement new therapies that facilitate clinical change. |  |  |  |  |  |  |
| Early intensive manual therapy can be broken down into manageable parts that can be implemented in stages. |  |  |  |  |  |  |
| I have timely access to data for monitoring and evaluation of therapies that I implement. This may include data from anecdotal feedback to validated outcome measures, on an individual or group level. |  |  |  |  |  |  |
| My workplace discusses quantitative and qualitative information about the success of implementation of new therapies. |  |  |  |  |  |  |
| My workplace discusses quantitative and qualitative information about the success of new therapies to determine whether desired outcomes are being achieved (e.g., 2 point increase on COPM [Canadian Occupational Performance Measure]). |  |  |  |  |  |  |
| Early intensive manual therapy can be adapted to an optimal fit and integrated into my workplace processes, while maintaining an intensive approach. |  |  |  |  |  |  |
| At my workplace, young children with a high probability for a CP diagnosis are referred for occupational therapy services before 1 years old. |  |  |  |  |  |  |
| At my workplace, young children with a high probability for a CP diagnosis receive occupational therapy services before 1 years old. |  |  |  |  |  |  |

Thank you for completing Part One. Do you have any feedback to share?

________________________________________________________

**PART TWO: Demographics**

Q1. How long have you been a practicing Occupational Therapist?

- Years __________________________________________________
- Months __________________________________________________

Q2. How long have you been providing therapy to children with cerebral palsy (CP)?

- Years __________________________________________________

Q3. Approximately how many hours a week do you spend treating children with CP?

- Hours __________________________________________________

Q4. Do you use the following service delivery models in your primary work setting?
 Select all that apply.

- 1:1 therapist to client ratio
- Partial supervision (one therapist, several clients)
- Group (structured activities in a group format)
- Other: __________________________________________________

Q5. Do you offer early intensive manual therapy (e.g., constraint induced movement therapy and/or bimanual therapy) for children with CP <2 years old?

- Yes
- No

Display This Question:

If Do you offer early intensive manual therapy (e.g., constraint induced movement therapy and/or bim... = Yes

Q6. How long have you been providing this therapy?

- Years __________________________________________________
- Months __________________________________________________

Display This Question:

If Do you offer early intensive manual therapy (e.g., constraint induced movement therapy and/or bim... = Yes

Q7. Do you follow a specific protocol (e.g., Baby-CIMT)?

- Yes: please specify __________________________________________________
- No

Display This Question:

If Do you offer early intensive manual therapy (e.g., constraint induced movement therapy and/or bim... = Yes

Q8. How long is the intensive program?

- Weeks __________________________________________________

Display This Question:

If Do you offer early intensive manual therapy (e.g., constraint induced movement therapy and/or bim... = Yes

Q9. How frequent is the practice?

- Daily
- 5-6 times per week
- 3-4 times per week
- 2 times per week
- Once per week

Display This Question:

If Do you offer early intensive manual therapy (e.g., constraint induced movement therapy and/or bim... = Yes

Q10. How long is each practice session?
Time can be distributed within the day (e.g., 15 mins + 15 mins).

- >30 minutes
- 30 minutes
- 20 minutes
- 15 minutes
- 10 minutes

Display This Question:

If Do you offer early intensive manual therapy (e.g., constraint induced movement therapy and/or bim... = Yes

Q11. What practice model does your team use for early intensive manual therapy?
Select all that apply.

- OT hands-on delivers in-person therapy in-home
- OT hands-on delivers in-person therapy in clinic
- OT delivers virtual sessions
- OT coaches caregiver to deliver therapy
- More than one OT or therapist assistant shares responsibility for therapy delivery for each child

Display This Question:

If Do you offer early intensive manual therapy (e.g., constraint induced movement therapy and/or bim... = No

Q12. Do you offer any other manual therapy for children with CP <2 years old?

- Yes. Please describe: __________________________________________________
- No.

Q13. In which Canadian provinces or territories are you currently licensed to practice? Check all that apply:

- Alberta
- British Columbia
- Manitoba
- New Brunswick
- Newfoundland and Labrador
- Northwest Territories
- Nova Scotia
- Nunavut
- Ontario
- Prince Edward Island
- Quebec
- Saskatchewan
- Yukon

Q14. Is a specialist referral required to obtain occupational therapy services in your workplace setting?
Select all that apply.

- Yes
- No
- Varies by funding
- Other: __________________________________________________

Q15. Are you the only occupational therapist proving manual therapy to children with CP at your workplace?

- Yes
- No

Q16. What is the age range of children with CP who you work with?
Select all that apply.

- 0 to
- 2 to 4 years old
- 5 to 8 years old
- 9 to 12 years old
- 13 to 18 years old

Q17. Have you received training on the following assessments/exams?
Select all that apply.

- Hand Assessments for Infants (HAI)
- Mini- Assisting Hand Assessments (mini-AHA)
- Assisting Hand Assessments (AHA)
- Hammersmith Infant Neurological Examination (HINE)

Q18. Do you travel to client homes to deliver therapy?

- Yes
- No

Q19. Do you coach caregivers to deliver hand therapy for their child with cerebral palsy?

- Yes
- No

Display This Question:

If Do you coach caregivers to deliver hand therapy for their child with cerebral palsy? = Yes

Q20. Do you travel to clients’ homes for coaching sessions?

- Yes
- No

Q21. At your workplace, what is the approximate timeline between receipt of a referral & first visit?

- Months __________________________________________________

Q22. Do you work at a:
Select all that apply.

- Private practice
- Tertiary centre
- Community practice
- School

Q23. At my workplace, therapy assistants provide early intensive manual therapy under the supervision of an OT.

- Yes
- No

Q24. What are the first three digits of your workplace postal code?

________________________________________________________________

*The remaining questions in Part Two are included to ensure that we capture diverse voices in this research. You have the option to select "Prefer not to answer" for these questions.*

Q25. What is the highest level of schooling you have obtained?

- Indigenous knowledge keeper
- Grade school certificate
- High school certificate
- College certificate or diploma
- Bachelor’s degree
- Master’s, doctorate, or professional (e.g., law, dentistry, pharmacy) degree
- Prefer not to answer

Q26. What language(s) are you comfortable speaking at your workplace?

- English
- French
- Other (please specify): __________________________________________________
- Prefer not to answer

Q27. Do you identify as a Racialized person?

- Yes
- No
- Prefer not to answer

Q28. Which of the following best describes you? 
Select all that apply.

- First Nations
- Métis
- Inuit
- Black
- East Asian (Chinese, Korean, Japanese)
- Latin American
- Middle Eastern (Arab, West Asian (e.g., Iranian, Afghan))
- South Asian (e.g., East Indian, Pakistani, Sri Lankan)
- Southeast Asian (Filipino, Vietnamese, Cambodian, Laotian, Thai)
- White
- Do not know
- Other please specify: __________________________________________________
- Prefer not to answer

Q29. Do you self-identify as a person with a disability?

- Yes
- No
- Prefer not to answer

Display This Question:

If Do you self-identify as a person with a disability? = Yes

Q30. Please indicate the types(s) of disability you have:

- Visible
- Non-visible
- Both
- Prefer not to answer

Q31. How do you identify with regards to your gender?
Select all that apply.

- Woman
- Man
- Non-Binary, Gender Queer, or a similar term
- Two-spirit
- Transwoman
- Transman
- Other (please specify): __________________________________________________
- I do not identify with a gender
- Prefer not to answer

Q32. How do you identify with regards to your sexual orientation?
Select all that apply.

- Heterosexual/Straight
- Asexual
- Bisexual
- Two-spirit
- Gay
- Lesbian
- Queer
- Other (please specify): __________________________________________________
- Prefer not to answer

Q33. Thank you for completing Part Two, which included questions about you.

Do you have any feedback about the questions in Part Two?
_____________________________________________________________

Q34. Thank you for participating in the survey. Please share any feedback here.

_____________________________________________________________
