# Supplementary material for: Facilitators and barriers to implementation of early intensive manual therapies for young children with cerebral palsy across Canada
Source: BMC Health Serv Res. 2025 Apr 4;25:503. doi: 10.1186/s12913-025-12621-z (PMC11971912; doi:10.1186/s12913-025-12621-z)
Supplement: Supplementary file 3 — Supplementary Material 3: Appendix 3. Healthcare administrator Survey Version. [file 12913_2025_12621_MOESM3_ESM.docx]

**Healthcare Administrator Survey Version**

Welcome to Part One.

In Part One of the survey, we ask about things that influence delivery of an intensive hand therapy program for children under 2 years old. We are focusing on hand therapy. This may be constraint induced movement therapy (CIMT), bimanual therapy, or another type of therapy thar focuses on the child’s weaker/less-preferred hand. An intensive therapy program involves practice daily or many times per week. Caregivers may deliver therapy, therapists may support caregivers, or a therapist may deliver therapy. Visits with OTs may be in-person, at a clinic or the child’s home, or virtual over video call.

Part One survey questions are statements that have six response options.

| **PART ONE: Statements** | Response | | | | | |
| --- | --- | --- | --- | --- | --- | --- |
|  | Strongly Disagree | Disagree | Neutral | Agree | Strongly Agree | I do not have the experience/ knowledge to comment |
| My workplace will only adopt an early intensive manual therapy protocol that was developed by an experienced team. |  |  |  |  |  |  |
| Early intensive manual therapy has robust evidence supporting its effectiveness, such as a systematic review. |  |  |  |  |  |  |
| Early intensive manual therapy results in greater improvements than any other available therapies. |  |  |  |  |  |  |
| Early intensive manual therapy can be adapted or modified to fit a diversity of settings, families, and delivery models. |  |  |  |  |  |  |
| The implementation of early intensive manual therapy can be piloted in my workplace before adoption, by introducing in parts or on a small scale. |  |  |  |  |  |  |
| Early intensive manual therapy requires specialized training. |  |  |  |  |  |  |
| Early intensive manual therapy protocols clearly define therapy components and required training materials. |  |  |  |  |  |  |
| Early intensive manual therapy is affordable, including costs to train therapists and deliver the intensive therapy. |  |  |  |  |  |  |
| The decisions of my high-level workplace leaders, for example a rehabilitation program manager, influence whether OTs in my workplace implements early intensive manual therapy. |  |  |  |  |  |  |
| The decisions of my mid-level workplace leaders, for example a team or clinical practice lead, influence whether my workplace implements early intensive manual therapy. |  |  |  |  |  |  |
| Informal leaders influence whether my workplace implements early intensive manual therapy. Note: an informal leader is someone who exerts influence through their authority and status or people who exert influence through their credibility. Examples include senior therapists, parent advocates, subject matter experts. |  |  |  |  |  |  |
| At my workplace, there is at least one person whose job description includes driving implementation of new therapies, and/or facilitating implementation decisions of any new therapies. |  |  |  |  |  |  |
| The people working to implement any new therapies include the therapists administering the therapy and the clients receiving it. |  |  |  |  |  |  |
| At my workplace, an OT or therapy assistant is expected to be the primary provider of hands-on early intensive manual therapy. |  |  |  |  |  |  |
| At my workplace, an OT or therapy assistant is expected to coach caregivers to be the primary providers of hands-on early intensive manual therapy. |  |  |  |  |  |  |
| OTs at my workplace have the necessary knowledge and skills for hands-on delivery of early intensive manual therapy. |  |  |  |  |  |  |
| Caregivers of children with CP have the necessary capacity and skills to deliver early intensive manual therapy. |  |  |  |  |  |  |
| OTs at my workplace have dedicated time to coach the caregivers to be the primary providers of hands-on early intensive manual therapy with recurring coaching sessions. (Note: This can include support from their team, e.g., another OT or therapist assistant completing some visits.) |  |  |  |  |  |  |
| OTs at my workplace have the time required to be the primary providers of hands-on early intensive manual therapy (Note: This can include support from their team, e.g., another OT or therapist assistant completing some visits.) |  |  |  |  |  |  |
| OTs at my workplace are fully motivated to provide early intensive manual therapy through hands-on delivery or coaching. |  |  |  |  |  |  |
| OTs at my workplace can easily travel to clients' homes for in-person sessions. |  |  |  |  |  |  |
| Some of the clients can’t easily travel to my workplace for in-person sessions. |  |  |  |  |  |  |
| OTs have reliable internet connections for virtual therapy. |  |  |  |  |  |  |
| Caregivers have reliable internet connections for virtual therapy. |  |  |  |  |  |  |
| My workplace requires the option of virtual therapy sessions. Reasons could include travel time/cost or workplace policy. |  |  |  |  |  |  |
| My workplace requires the option of therapy to be delivered in-person either in a hospital or clinic site, or in family homes. |  |  |  |  |  |  |
| I have strong professional relationships with my workplace team and leaders. |  |  |  |  |  |  |
| New ideas for therapy are valued by my workplace team and leaders. |  |  |  |  |  |  |
| My team has effective communication methods with caregivers, like access to interpreters and written materials in multiple languages. |  |  |  |  |  |  |
| My workplace uses data to inform continual improvement. |  |  |  |  |  |  |
| I can share recommendations/ ideas for new projects or changes in procedures to my leader(s). |  |  |  |  |  |  |
| My workplace’s current occupational therapy practice for young children with CP needs to change. |  |  |  |  |  |  |
| Early intensive manual therapy fits well within my workplace/workflow. |  |  |  |  |  |  |
| Implementing early intensive manual therapy is a top priority at my workplace. |  |  |  |  |  |  |
| Funding is available for early intensive manual therapy at my workplace. |  |  |  |  |  |  |
| My workplace has appropriate physical space for hands-on delivery of early intensive manual therapy. |  |  |  |  |  |  |
| Some of my clients don’t have appropriate physical space to deliver early intensive manual therapy in their homes. |  |  |  |  |  |  |
| My workplace has appropriate seating and toys for hands-on delivery of early intensive manual therapy. |  |  |  |  |  |  |
| Most clients have appropriate seating and toys to deliver early intensive manual therapy in their homes. |  |  |  |  |  |  |
| OTs at my workplace have the opportunity to access mentorship and training for hands-on delivery of early intensive manual therapy. |  |  |  |  |  |  |
| OTs at my workplace have the opportunity to access mentorship and training to coach caregivers to be the primary provider of hands-on early intensive manual therapy. |  |  |  |  |  |  |
| OTs at my workplace have received adequate training and/or mentorship to integrate clients' cultural beliefs, values and practices. |  |  |  |  |  |  |
| OTs at my workplace are able to provide a modified approach to early intensive manual therapy in the event of a critical incident, like a global pandemic or change in political leadership. |  |  |  |  |  |  |
| My workplace is in a community that supports early intensive manual therapy. This includes social and economic factors, such as attitudes towards therapy and financial/time resources to attend therapy. |  |  |  |  |  |  |
| My workplace is connected with referral networks, for example between health and social services. |  |  |  |  |  |  |
| My workplace has academic partnerships, for example with an academic institution or with a healthcare centre affiliated with an academic institution. |  |  |  |  |  |  |
| The implementation of early intensive manual therapy is influenced by my workplace policies or procedures. |  |  |  |  |  |  |
| My workplace requires external grants or reimbursement (e.g., private health insurance or Jordan’s Principle) to implement early intensive manual therapy. |  |  |  |  |  |  |
| My team collaborates to implement new therapies. |  |  |  |  |  |  |
| When implementing new therapies, my workplace considers the priorities, preferences and needs of OTs. |  |  |  |  |  |  |
| When implementing new therapies, my workplace considers the priorities, preferences and needs of caregivers. |  |  |  |  |  |  |
| My workplace collects information to identify and appraise barriers and facilitators before implementation and delivery of a new therapy. |  |  |  |  |  |  |
| My workplace systematically plans implementation of new therapies in advance. |  |  |  |  |  |  |
| My workplace uses a defined implementation strategy that considers barriers, facilitators and outcome measures when implementing a new therapy. |  |  |  |  |  |  |
| My workplace encourages OTs to plan and implement new therapies that facilitate clinical change. |  |  |  |  |  |  |
| When implementing new therapies, my workplace considers the priorities, preferences and needs of caregivers. |  |  |  |  |  |  |
| Early intensive manual therapy can be broken down into manageable parts that can be implemented in stages. |  |  |  |  |  |  |
| I have timely access to data for monitoring and evaluation of OT therapies. This may include data from anecdotal feedback to validated outcome measures, on an individual or group level. |  |  |  |  |  |  |
| My workplace analyzes quantitative data and qualitative feedback to make decisions about the success of implementation of new therapies, and whether desired outcomes are being achieved. |  |  |  |  |  |  |
| Early intensive manual therapy can be adapted to an optimal fit and integrated into my workplace processes, while maintaining an intensive approach. |  |  |  |  |  |  |
| At my workplace, young children with a high probability for a CP diagnosis are referred for occupational therapy services before 1 years old. |  |  |  |  |  |  |
| At my workplace, young children with a high probability for a CP diagnosis receive occupational therapy services before 1 years old. |  |  |  |  |  |  |

Thank you for completing Part One. Do you have any feedback to share?

________________________________________________________

**PART TWO: Demographics**

Q1. What is your title in your current role (e.g., rehabilitation manager, team lead)?

________________________________________________________________

Q2. What type of decisions do you make in your role?
Select all that apply.

- I am involved in budgetary decisions.
- I am involved in higher level program direction and strategic planning.
- I am involved in therapist resource allocation decisions.
- I am involved in best-evidence practice decisions and planning.
- I am involved in therapist practice, competency, and advancement of clinical services.
- I am involved in integration of relevant research, evaluation and clinical guidelines into practice.
- I am involved in coordinating day to day operations of clinical practice.
- Other (please specify): __________________________________________________

Q3. How long have you been in your role?

- Years __________________________________________________
- Months __________________________________________________

Q4. How many OTs are in your program?

- Number of OTs __________________________________________________

Q5. Do OTs in your program use the following service delivery models?
 Select all that apply.

- 1:1 therapist to client ratio
- Partial supervision (one therapist, several clients)
- Group (structured activities in a group format)
- Other: __________________________________________________

Q6. Do OTs in your program offer early intensive manual therapy (e.g., constraint induced movement therapy and/or bimanual therapy) for children with CP <2 years old?

- Yes
- No

Display This Question:

If Do OTs in your program offer early intensive manual therapy (e.g., constraint induced movement th... = Yes

Q7. How long has your team been providing this therapy?

- Years __________________________________________________
- Months __________________________________________________

Display This Question:

If Do OTs in your program offer early intensive manual therapy (e.g., constraint induced movement th... = Yes

Q8. Does your team follow a specific protocol (e.g., Baby-CIMT)?

- Yes. Please specify: __________________________________________________
- No
- Unsure

Display This Question:

If Do OTs in your program offer early intensive manual therapy (e.g., constraint induced movement th... = Yes

Q9. How long is the intensive program?

- Weeks __________________________________________________
- Unsure

Display This Question:

If Do OTs in your program offer early intensive manual therapy (e.g., constraint induced movement th... = Yes

Q10. How frequent is the practice?

- Daily
- 5-6 times per week
- 3-4 times per week
- 2 times per week
- Once per week
- Unsure

Display This Question:

If Do OTs in your program offer early intensive manual therapy (e.g., constraint induced movement th... = Yes

Q11. How long is each practice session?
Time can be distributed within the day (e.g., 15 mins + 15 mins).

- >30 minutes
- 30 minutes
- 20 minutes
- 15 minutes
- 10 minutes
- Unsure

Display This Question:

If Do OTs in your program offer early intensive manual therapy (e.g., constraint induced movement th... = Yes

Q12. What practice model does your team use for early intensive manual therapy?
Select all that apply.

- OT hands-on delivers in-person therapy in-home
- OT hands-on delivers in-person therapy in clinic
- OT delivers virtual sessions
- OT coaches caregiver to deliver therapy
- More than one OT or therapist assistant shares responsibility for therapy delivery for each child

Display This Question:

If Do OTs in your program offer early intensive manual therapy (e.g., constraint induced movement th... = No

Q13. Does your team offer any other manual therapy for children with CP <2 years old?

- Yes. Please describe: __________________________________________________
- No.

Q14. Is a specialist referral required to obtain occupational therapy services in your workplace setting?
Select all that apply.

- Yes
- No
- Varies by funding
- Other: __________________________________________________

Q15. At your workplace, what is the approximate timeline between receipt of a referral & first visit?

- Months __________________________________________________

Q16. At my workplace, therapy assistants provide early intensive manual therapy under the supervision of an OT.

- Yes
- No

Q17. Do you work at a:
Select all that apply.

- Private practice
- Tertiary centre
- Community practice
- School

Q18. What are the first three digits of your workplace postal code?

________________________________________________________________

*The remaining questions in Part Two are included to ensure that we capture diverse voices in this research. You have the option to select "Prefer not to answer" for these questions.*

Q19. What is the highest level of schooling you have obtained?

- College certificate or diploma
- Bachelor’s degree
- Master’s
- Doctorate
- Prefer not to answer

Q20. What language(s) are you comfortable speaking at your workplace?

- English
- French
- Other (please specify): __________________________________________________
- Prefer not to answer

Q21. Do you identify as a Racialized person?

- Yes
- No
- Prefer not to answer

Q22. Which of the following best describes you? 
Select all that apply.

- First Nations
- Métis
- Inuit
- Black
- East Asian (Chinese, Korean, Japanese)
- Latin American
- Middle Eastern (Arab, West Asian (e.g., Iranian, Afghan))
- South Asian (e.g., East Indian, Pakistani, Sri Lankan)
- Southeast Asian (Filipino, Vietnamese, Cambodian, Laotian, Thai)
- White
- Do not know
- Other please specify: __________________________________________________
- Prefer not to answer

Q23. Do you self-identify as a person with a disability?

- Yes
- No
- Prefer not to answer

Display This Question:

If Do you self-identify as a person with a disability? = Yes

Q24. Please indicate the types(s) of disability you have:

- Visible
- Non-visible
- Both
- Prefer not to answer

Q25. How do you identify with regards to your gender?
Select all that apply.

- Woman
- Man
- Non-Binary, Gender Queer, or a similar term
- Two-spirit
- Transwoman
- Transman
- Other (please specify): __________________________________________________
- I do not identify with a gender
- Prefer not to answer

Q26. How do you identify with regards to your sexual orientation?
Select all that apply.

- Heterosexual/Straight
- Asexual
- Bisexual
- Two-spirit
- Gay
- Lesbian
- Queer
- Other (please specify): __________________________________________________
- Prefer not to answer

Q27. Thank you for completing Part Two, which included questions about you.

Do you have any feedback about the questions in Part Two?
_____________________________________________________________

Q28. Thank you for participating in the survey. Please share any feedback here.

_____________________________________________________________
