# Supplementary figures and images for: Facilitators and barriers to implementation of early intensive manual therapies for young children with cerebral palsy across Canada
Source: BMC Health Serv Res. 2025 Apr 4;25:503. doi: 10.1186/s12913-025-12621-z (PMC11971912; doi:10.1186/s12913-025-12621-z)

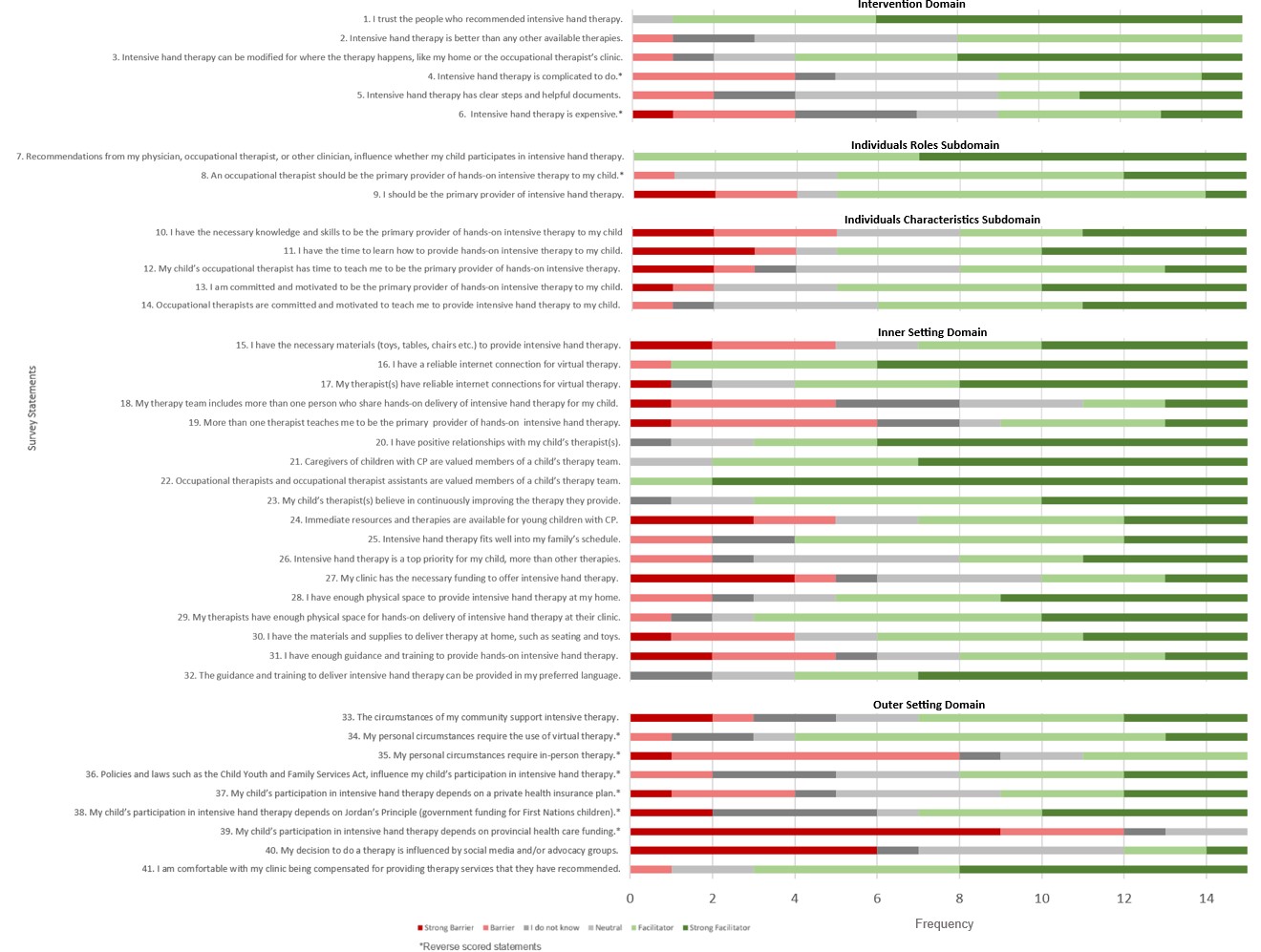

Supplement: Supplementary file 4 — Supplementary Material 4: Appendix 4. Frequency Diagram of Caregiver Survey Responses. [file 12913_2025_12621_MOESM4_ESM.jpg]

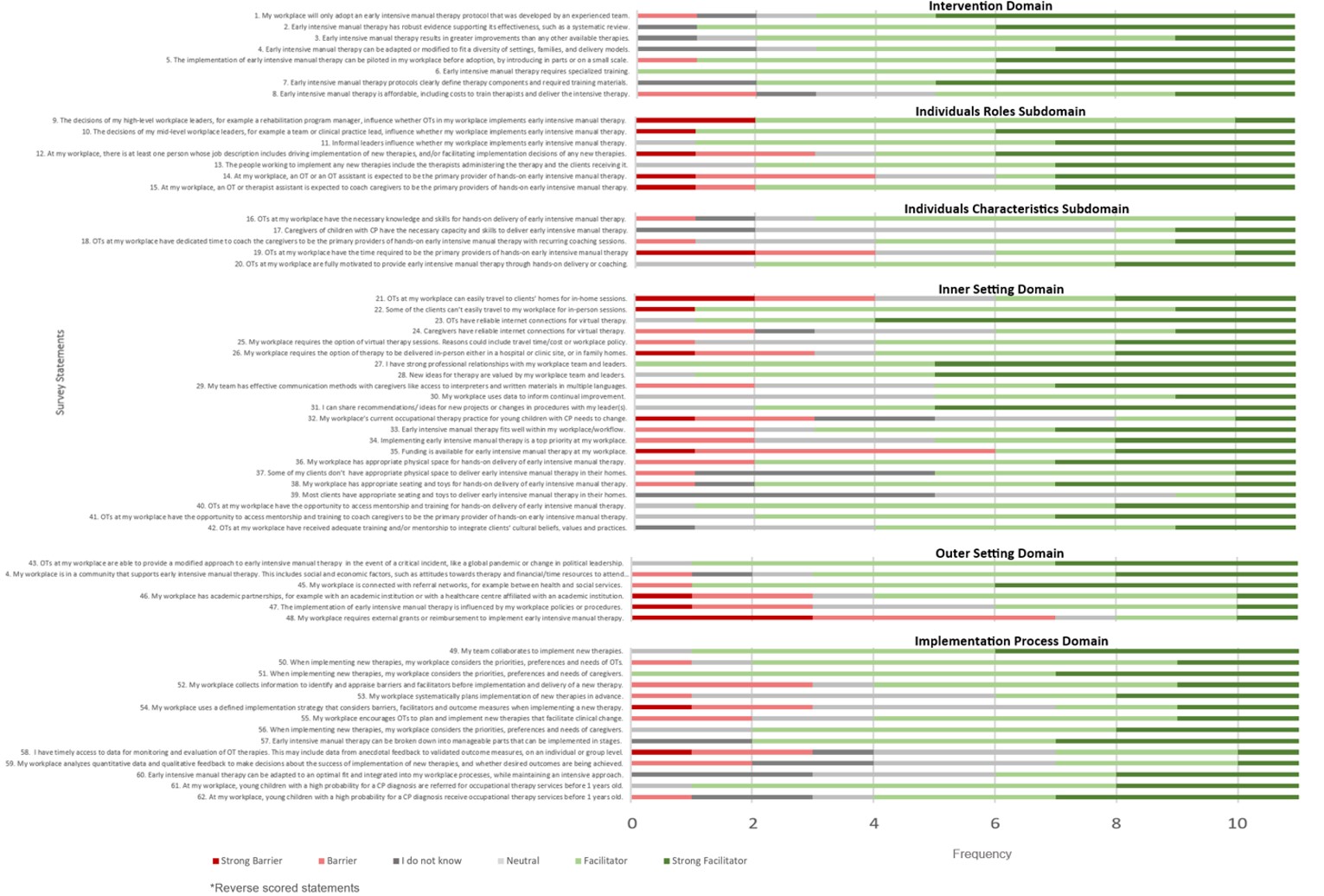

Supplement: Supplementary file 5 — Supplementary Material 5: Appendix 5. Frequency Diagram of Healthcare Administrators Survey Responses. [file 12913_2025_12621_MOESM5_ESM.jpg]

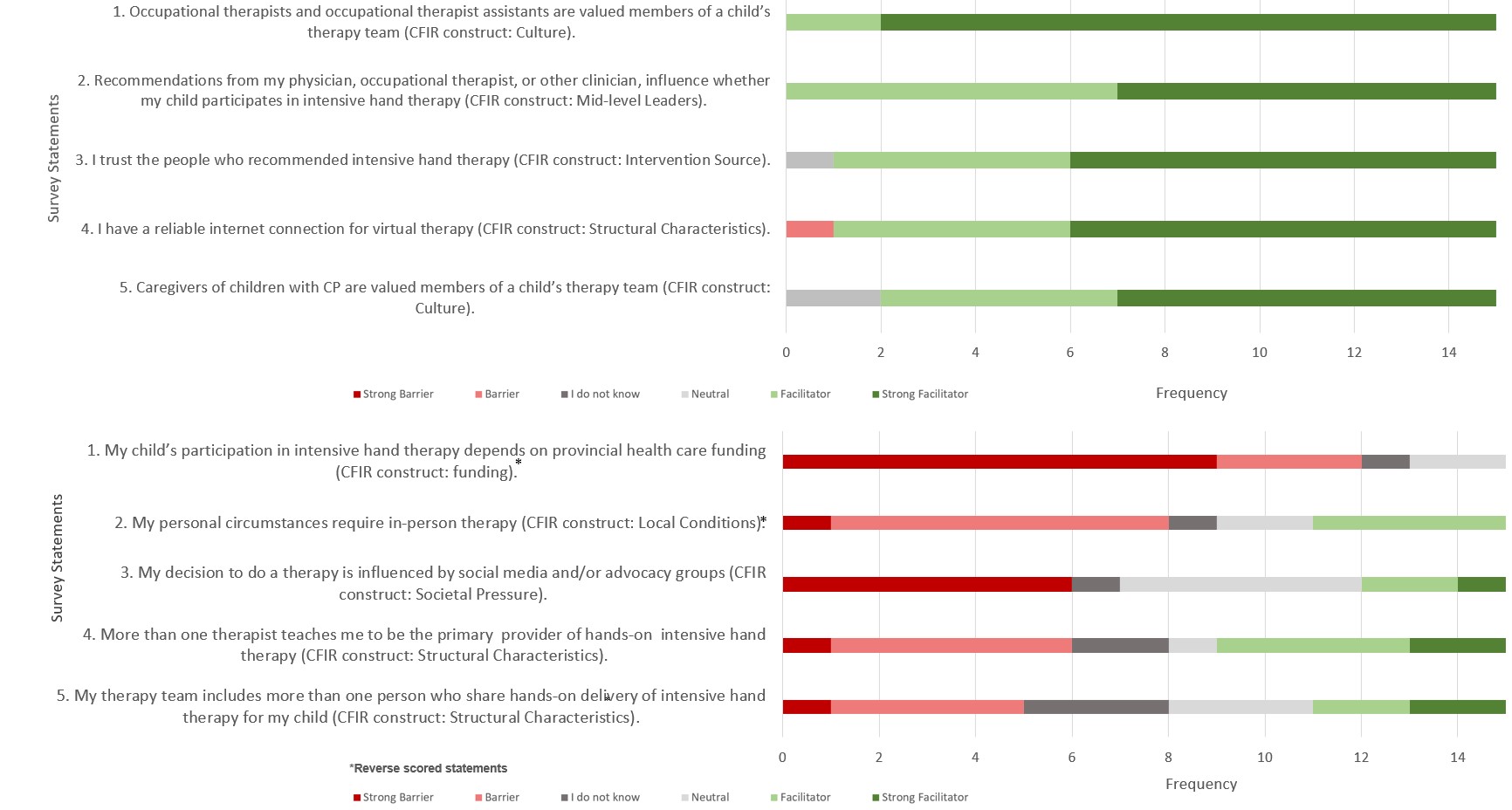

Supplement: Supplementary file 6 — Supplementary Material 6: Appendix 6. Frequency diagram of Caregiver respondents (n = 15) of top five most frequently endorsed facilitators (top) and barriers (bottom) to implementation reported. [file 12913_2025_12621_MOESM6_ESM.jpg]

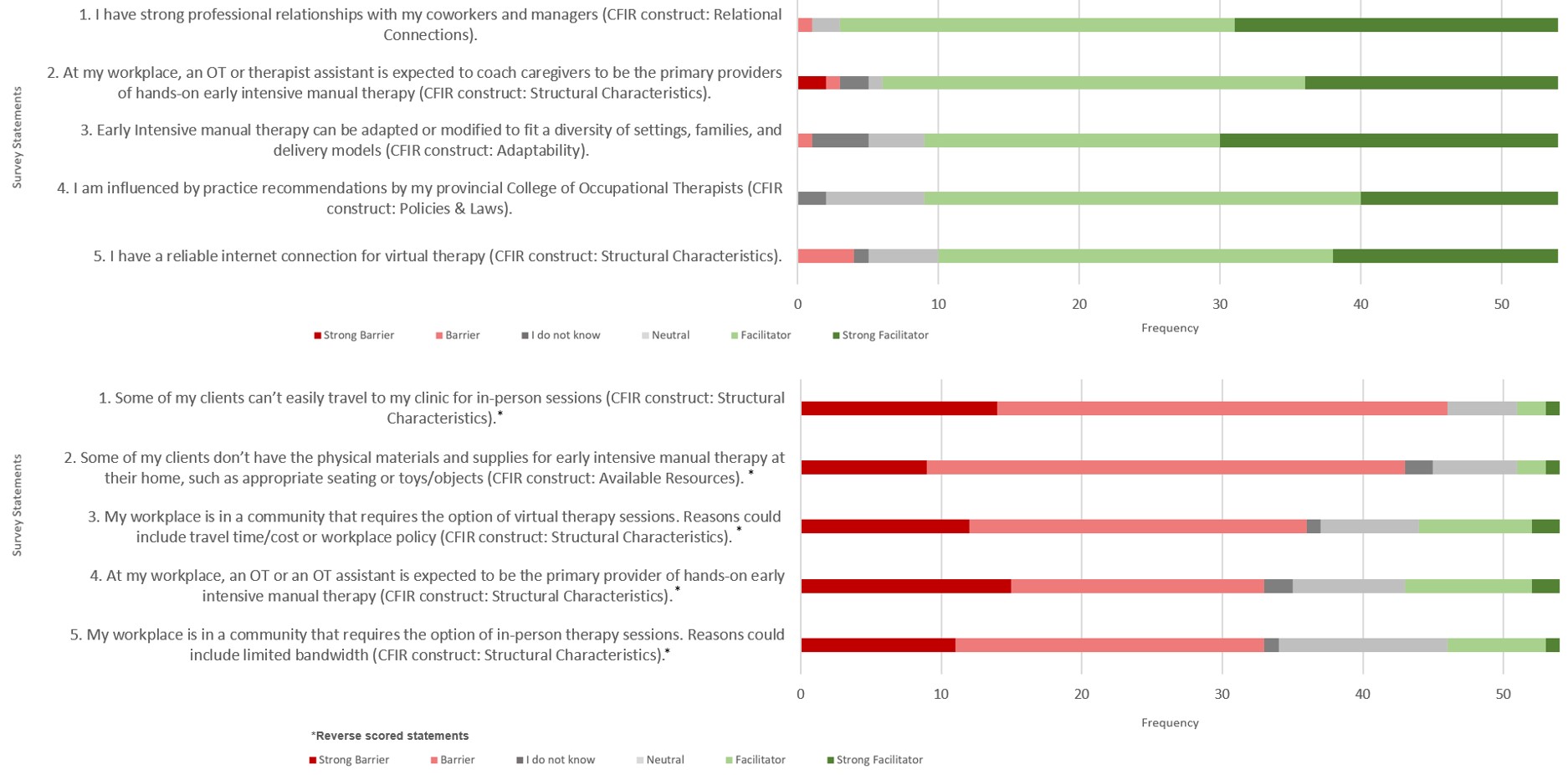

Supplement: Supplementary file 7 — Supplementary Material 7: Appendix 7. Frequency diagram of OT respondents (n = 54) of top five most frequently endorsed facilitators (top) and barriers (bottom) to implementation reported. [file 12913_2025_12621_MOESM7_ESM.jpg]

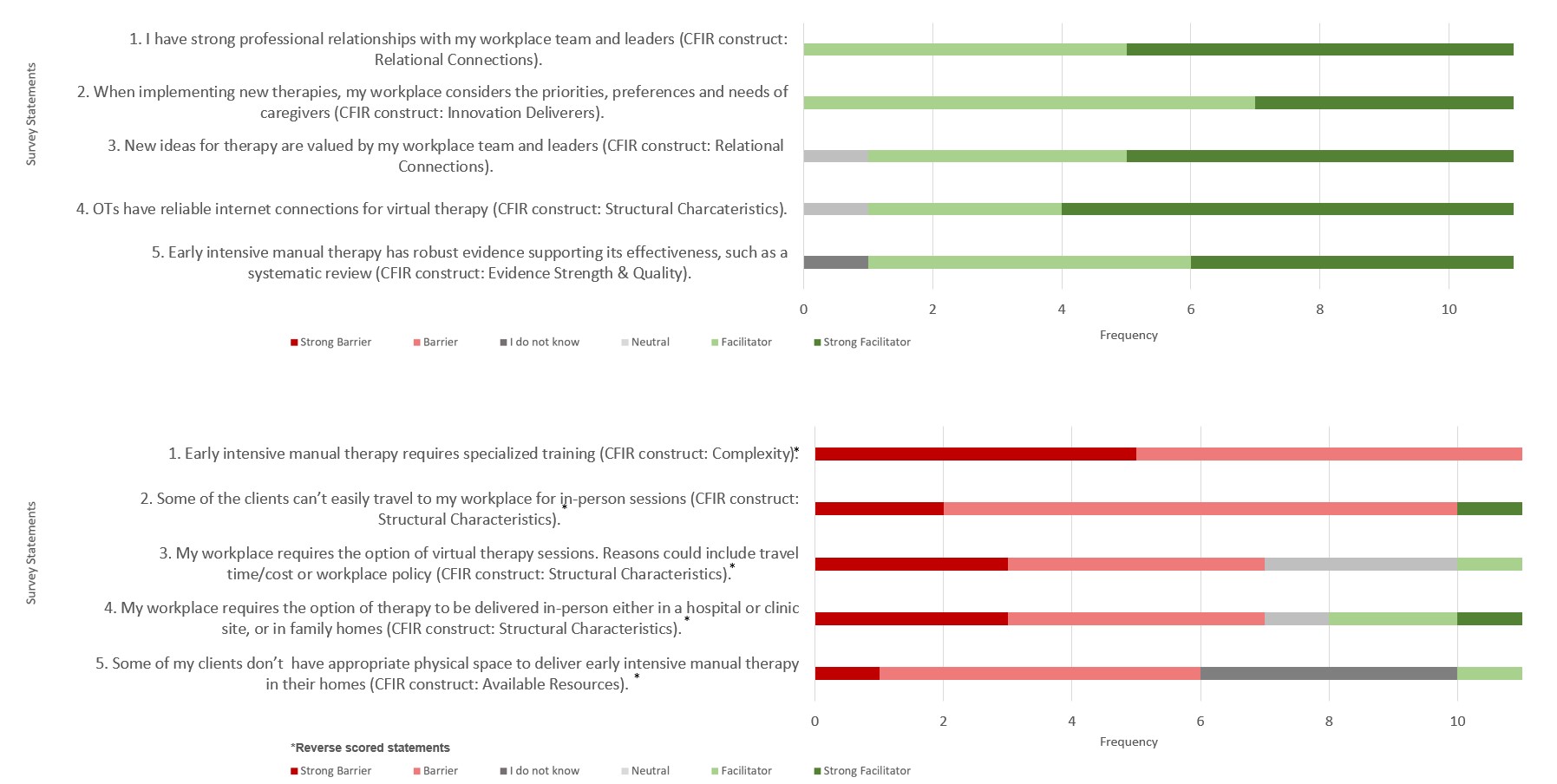

Supplement: Supplementary file 8 — Supplementary Material 8: Appendix 8. Frequency diagram of healthcare administrator respondents (n = 11) of top five most frequently endorsed facilitators (top) and barriers (bottom) to implementation reported. [file 12913_2025_12621_MOESM8_ESM.jpg]
