# Supplementary material for: Facilitators and barriers to implementation of early intensive manual therapies for young children with cerebral palsy across Canada
Source: BMC Health Serv Res. 2025 Apr 4;25:503. doi: 10.1186/s12913-025-12621-z (PMC11971912; doi:10.1186/s12913-025-12621-z)
Supplement: Supplementary file 9 — Supplementary Material 9: Appendix 9. Results from the PPEET Survey (n = 4 Caregivers, n = 3 OTs, n = 1 OT clinical practice lead). [file 12913_2025_12621_MOESM9_ESM.docx]

|  |  | **Strongly Disagree (score = 1)**  **n (%)** | **Disagree (score =2)**  **n (%)** | **Neither Disagree nor Agree**  **(score =3)**  **n (%)** | **Agree**  **(score =4)**  **n (%)** | **Strongly Agree**  **(score =5)**  **n (%)** |
| --- | --- | --- | --- | --- | --- | --- |
| PART A: Communication and support for participation | I have a clear understanding of the purpose of the study. | - | - | - | 3 (37.5) | 5 (62.5) |
|  | The supports I need to participate as a research partner are available (e.g., compensation for my time, childcare) | - | - | 1 (12.5) | 2 (25) | 5 (62.5) |
|  | I have enough information to carry out my role. | - | - | - | 3 (37.5) | 5 (62.5) |
| PART B: Sharing your views and perspectives | I am able to express my views freely. | - | - |  | 2 (25.0) | 6 (75.0) |
|  | I feel that my views are heard. | - | - | 1 (12.5) | 1 (12.5) | 6 (75.0) |
|  | A wide range of views on discussion topics is shared. | - | - | 1 (12.5) | 1 (12.5) | 6 (75.0) |
|  | The individuals participating in the research study team represent a broad range of perspectives. | - | - | - | 1 (12.5) | 7 (87.5) |
| PART C: Impacts and influence of the engagement initiative | The research project is achieving its stated objectives. | - | - | 1 (12.5) | 2 (25.0) | 5 (62.5) |
|  | I am confident that the research team takes feedback provided by partners into consideration. | - | - | - | 3 (37.5) | 5 (62.5) |
|  | I think that the work of partners makes a difference to the work of the research study. | - | - | - | 3 (37.5) | 5 (62.5) |
| PART D: Final thoughts | As a result of my participation in the research study, I am better informed about engagement in research. | - | - | 1 (12.5) | 3 (37.5) | 4 (50.0) |
|  | Overall, I am satisfied with this engagement initiative. | - | - | 1 (12.5) | 2 (25.0) | 5 (62.5) |
|  | This engagement initiative is a good use of my time. | - | - | - | 4 (50.0) | 4 (50.0) |
